# Late-onset Alzheimer’s disease is associated with inherent changes in bioenergetics profiles

**DOI:** 10.1038/s41598-017-14420-x

**Published:** 2017-10-25

**Authors:** Kai-C. Sonntag, Woo-In Ryu, Kristopher M. Amirault, Ryan A. Healy, Arthur J. Siegel, Donna L. McPhie, Brent Forester, Bruce M. Cohen

**Affiliations:** 1 Department of Psychiatry, McLean Hospital, Harvard Medical School, Belmont, MA 02478 USA; 2 Basic Neuroscience Division, McLean Hospital, Harvard Medical School, Belmont, MA 02478 USA; 3 Program for Neuropsychiatric Research, McLean Hospital, Harvard Medical School, Belmont, MA 02478 USA; 4 Internal Medicine Department, McLean Hospital, Harvard Medical School, Belmont, MA 02478 USA; 5 Mood Disorders Division and Geriatric Psychiatry Research Program, McLean Hospital, Harvard Medical School, Belmont, MA 02478 USA

## Abstract

Body-wide changes in bioenergetics, i.e., energy metabolism, occur in normal aging and disturbed bioenergetics may be an important contributing mechanism underlying late-onset Alzheimer’s disease (LOAD). We investigated the bioenergetic profiles of fibroblasts from LOAD patients and healthy controls, as a function of age and disease. LOAD cells exhibited an impaired mitochondrial metabolic potential and an abnormal redox potential, associated with reduced nicotinamide adenine dinucleotide metabolism and altered citric acid cycle activity, but not with disease-specific changes in mitochondrial mass, production of reactive oxygen species, transmembrane instability, or DNA deletions. LOAD fibroblasts demonstrated a shift in energy production to glycolysis, despite an inability to increase glucose uptake in response to IGF-1. The increase of glycolysis and the abnormal mitochondrial metabolic potential in LOAD appeared to be inherent, as they were disease- and not age-specific. Our findings support the hypothesis that impairment in multiple interacting components of bioenergetic metabolism may be a key mechanism contributing to the risk and pathophysiology of LOAD.

## Introduction

Alzheimer’s disease (AD) is an age-related neurodegenerative disorder characterized by slow progressive deterioration and death of neurons. A number of interacting factors determine the risk of AD and among the better-studied pathophysiologic pathways, the “amyloid cascade hypothesis” proposes that AD is precipitated by an accumulation of Aβ-containing plaques and tangles of hyperphosphorylated tau (p-tau)^1–3^. This hypothesis is best supported for familial/early-onset forms of AD (EOAD), while less so for the more common sporadic/late-onset forms (LOAD)^4,5^. In LOAD, accumulation of toxic Aβ and p-tau may not be the initial cause of neural degeneration and may instead be consequences of other causative factors^5,6^. Changes in bioenergetics, i.e., energy metabolism, are part of the normal aging process and disturbed bioenergetics may be a contributing mechanism underlying LOAD^7–10^. These anomalies are body-wide, but affect the brain most substantially because of its exceptionally high-energy requirements. Thus, changes of bioenergetics and metabolism could be at the core of determining the survival capacities of brain cells with age and under stress, with these processes influenced, in turn, by genetic predisposition, epigenetics, environment, and lifestyle.

Bioenergetics is the metabolism of various fuel molecules to produce and utilize energy through glycolysis, mitochondrial respiration, that is, oxidative phosphorylation (OxPhos), or the pentose phosphate pathway (PPP). Healthy eukaryotic cells produce ATP about 12% through glycolysis and 88% through OxPhos, on average. In the 1920s, German physician-chemist Otto Warburg discovered that mammalian cancer cells can switch from OxPhos to glycolysis when exposed to low oxygen, called the “Warburg effect”^11^. Unlike proliferating cells, post-mitotic neurons have very little ability to use glycolysis, as they lack key enzymes in the glycolytic pathway, e.g., there is evidence that neurons have very low levels of 6-phosphofructo-2-kinase/fructose-2,6-biphosphatase 3 (PFKFB3), a key enzyme in the glycolytic pathway, due to heightened degradation through the ubiquitin proteasome system (UPS)^12,13^. Thus, neurons don’t ordinarily make adaptive and compensatory shifts to glycolysis, including under stress conditions. Instead, they have a high demand for other oxidative substrates and, in particular, lactate, which is largely provided by proliferating astrocytes through their high glycolytic capacity. While aging neurons exhibit an “inverse Warburg effect”, with an increase in OxPhos, probably due to mitochondrial damage and inefficiency, astrocytes exhibit a compensatory “Warburg effect” by elevating their glycolysis to provide and meet the increased neuronal demand for lactate^14–18^. Accumulating abnormalities in glucose/lactate balance in combination with accelerated mitochondrial damage in neurons may be at the core of many cellular events in senescence and neurodegeneration, including the initiation and progression of LOAD^15^.

While the underlying causes of bioenergetic dysfunctions in aging and degenerating cells may be in part due to an increase in mitochondrial damage and dysfunction, deficiencies in glucose processing and glycolysis, including age-related resistance to insulin signaling, which has been implicated as a mechanism in senescence and neurodegeneration, may also contribute^10,19^. Here, we investigated whether bioenergetic changes occur in cells from LOAD patients and how these changes may relate to normal aging. Using a set of skin fibroblast lines, we show that LOAD cells have respiratory deficiencies and exhibit a Warburg-type effect that is disease-specific and unrelated to age, suggesting that bioenergetic abnormalities may be an inherent mechanism underlying LOAD.

## Results

### Profiling Bioenergetics with Seahorse Technology

**Figure 1:**
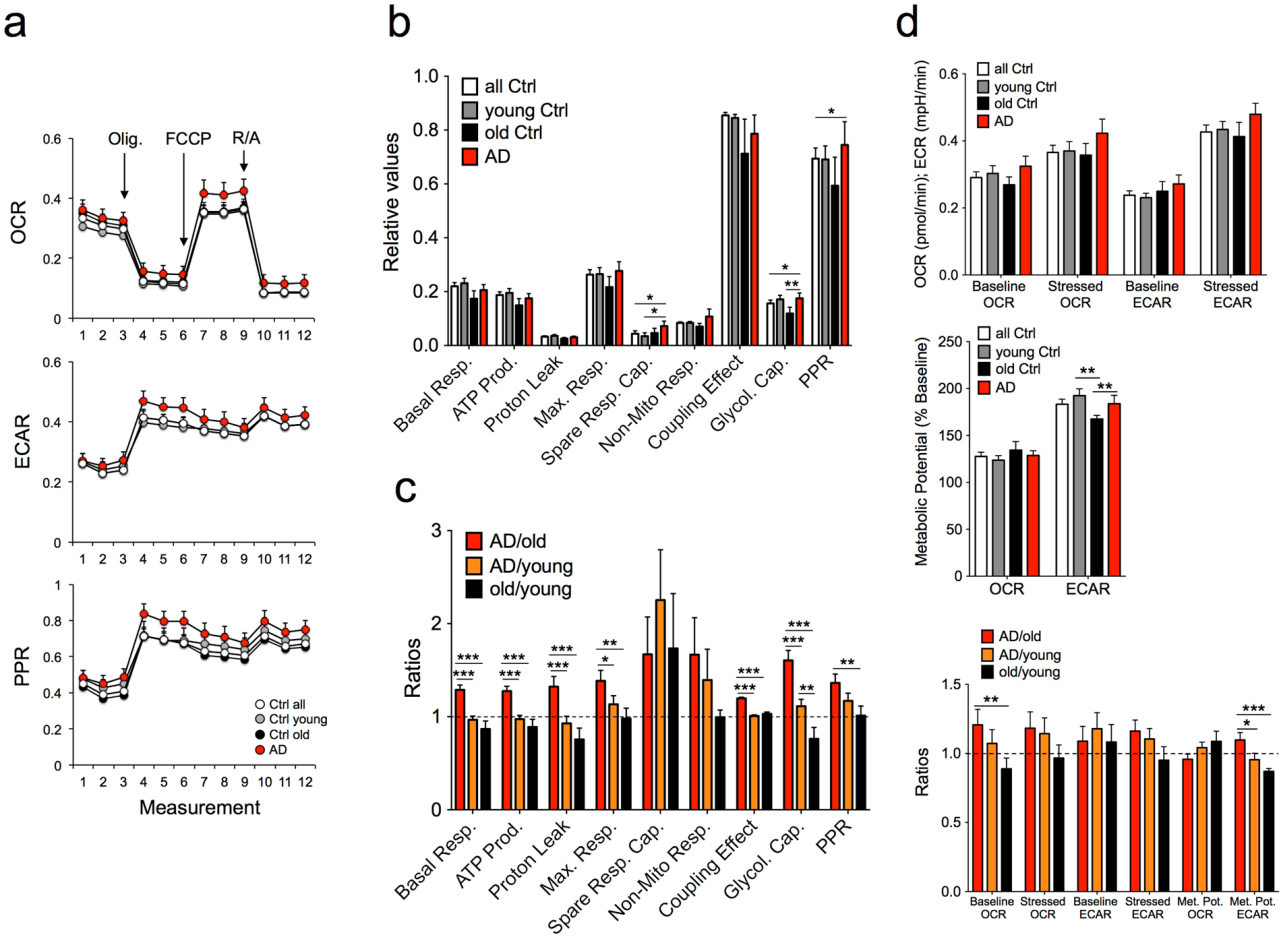
Data summary of Seahorse XFp Cell Mito Stress Test assays. (**a**) Profiles of Mito Stress Test data for OCR, ECAR, and PPR in *n* = 10 LOAD and *n* = 20 control fibroblast cell lines with arrows indicating injections into media of the specific stressors oligomycin (Olig.), carbonyl cyanite-4 (trifluoromethoxy) phenylhydrazone (FCCP), and Rotenone/Antimycin A (R/A) (see Supplementary Fig. 1 for additional details). (**b**) Relative values of parameters and (**c**) calculated ratios between healthy old versus young control, and LOAD versus young or old control samples. (**d**) Results from the Seahorse XF Cell Energy Phenotype Test Report Generator shown for OCR (pmol/min), ECAR (mpH/min), and metabolic potential ((stressed OCR or ECAR/baseline OCR or ECAR) × 100%)), and calculated ratios between old versus young control, and LOAD versus young or old control samples. Experiments were performed in *n* = 3 replicates. *P < 0.1; **P < 0.05; ***P < 0.01 (detailed statistical data can be found in Supplementary Table 2).

**Figure 2:**
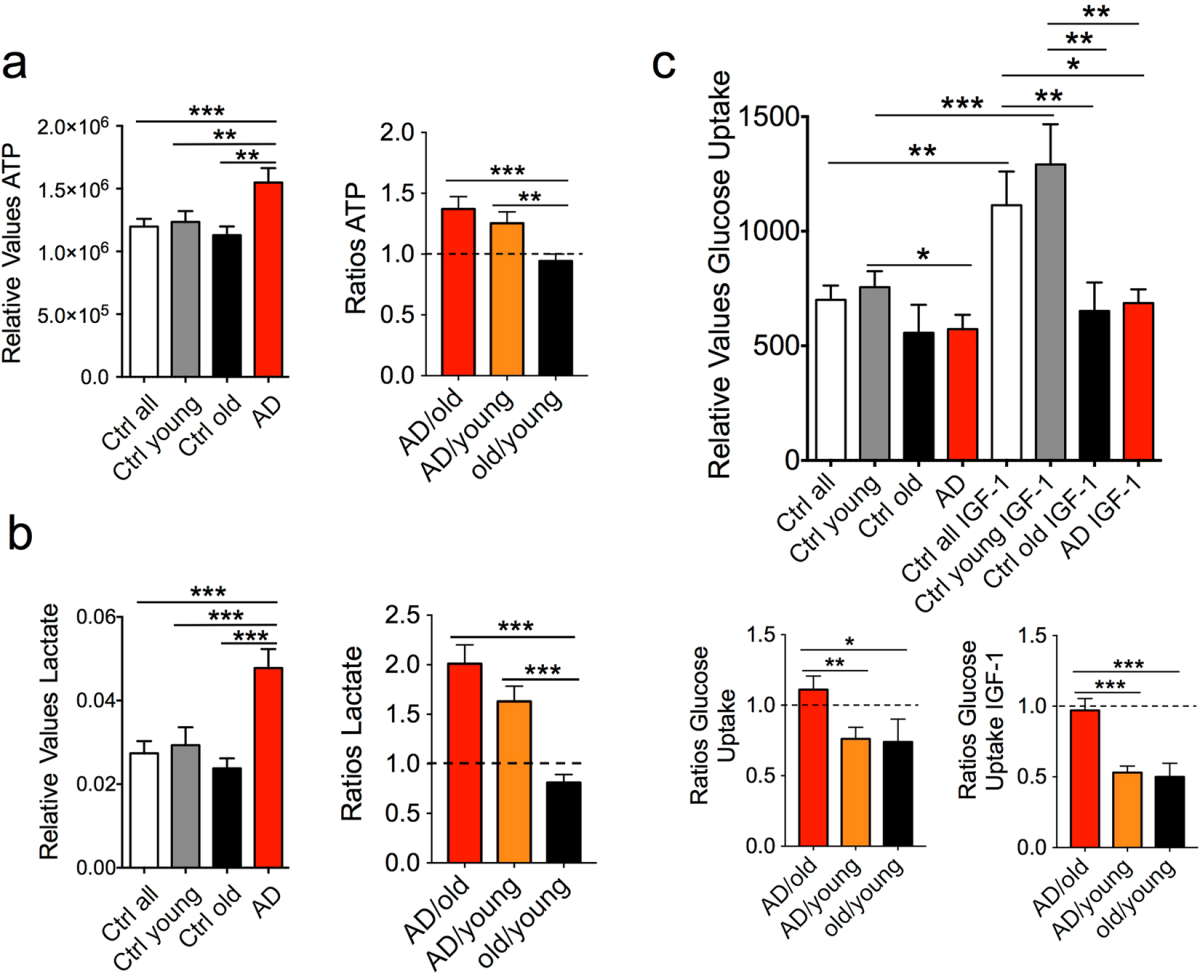
Bioenergetics parameters related to energy production and glycolysis. ATP (**a**) and L-Lactate (**b**) production were increased in LOAD fibroblasts when compared to controls. Glucose uptake in absence or presence of IGF-1 was decreased in LOAD and old fibroblasts when compared to young cells (**c**). Shown are relative values and ratios comparing AD with healthy old and young and healthy old with young samples. Two experiments were performed in *n* = 3 replicates. *P < 0.1; **P < 0.05; ***P < 0.01 (detailed statistical data are provided in Supplementary Table 2).

Experiments were performed on a total of 30 skin fibroblast cell lines divided into younger (age 21–54, *n* = 13) and older controls (age 55–75, *n* = 7), split according to the age range of the LOAD samples (ages 56–82, *n* = 10) (Supplementary Table 1). To measure parameters related to mitochondrial function and glycolysis we first used the Seahorse XFp Cell Mito Stress Test (Supplementary Fig. 1). The tests showed that the oxygen consumption rate (OCR), extracellular acidification rate (ECAR), and proton production rate (PPR) profiles were increased in LOAD fibroblasts when compared to all, young or old, controls (Fig. 1a). Calculated ratios derived from Seahorse XF analyzed values demonstrated a significant increase of basal respiration, ATP production, proton leak, maximal respiration, coupling effect, PPR, and glycolytic capacity in AD samples compared to young or old controls relative to ratios comparing old with young controls (Fig. 1b,c). Also, the ratios for non-mitochondrial respiration were increased in AD cells, although these differences were not statistically significant. In contrast, the ratios for basal respiration, ATP production, proton leak, maximal respiration, non-mitochondrial respiration, coupling effect, PPR, and glycolytic capacity appeared to be decreased in old fibroblasts compared to young or LOAD cells. To gain further insight into the bioenergetic profiles of our sample population, we additionally conducted a “Cell Energy Phenotype Test” provided by the Seahorse software. This test evaluates the metabolic potentials for OCR and ECAR by calculating changes after stress conditions relative to baseline. The test demonstrated an increase in baseline and stress levels for both OCR and ECAR in LOAD cells compared to young or old controls (Fig. 2d). In addition, there was a significant increase of the ECAR but no change in the OCR metabolic potential in LOAD cells when compared to old fibroblasts. Altogether, these data indicate that AD cells exhibit an increase in glycolysis, and show features of heightened mitochondrial activity, but their overall mitochondrial metabolic potential is impaired.

### Bioenergetics Parameters Related to Energy Production and Glycolysis

The increase of glycolytic capacities in LOAD fibroblasts observed in the Seahorse experiments suggests that these cells may have converted part of their energy production from OxPhos to glycolysis (Warburg effect). To assess this in more detail, we first measured the cells’ actual ATP levels, because the Seahorse assay indirectly measures ATP production by inhibiting mitochondrial complex V (see Supplementary Fig. 1). Consistent with the Seahorse-derived data, there was an increase of ATP production in LOAD samples when compared to all, young or old, controls (Fig. 2a). We then assessed L-Lactate production, as an indicator of glycolytic activity, and found elevated levels of lactate in AD fibroblasts compared to control cells (Fig. 2b). Interestingly, both ATP and L-Lactate production were not increased in healthy old versus young fibroblasts, indicating that these effects were specific to LOAD, not aging. In addition, we performed glucose uptake assays in combination with IGF-1, because IGF-1 promotes uptake of glucose and resistance to IGF-1/insulin signaling has been implicated as a factor in AD pathogenesis^10,19^. Both glucose uptake and the effects of IGF-1 were significantly reduced in old and LOAD fibroblast when compared to young cells, but were not different between old and LOAD cells (Fig. 2c).

### Parameters Related to Mitochondrial Function and Integrity

**Figure 3:**
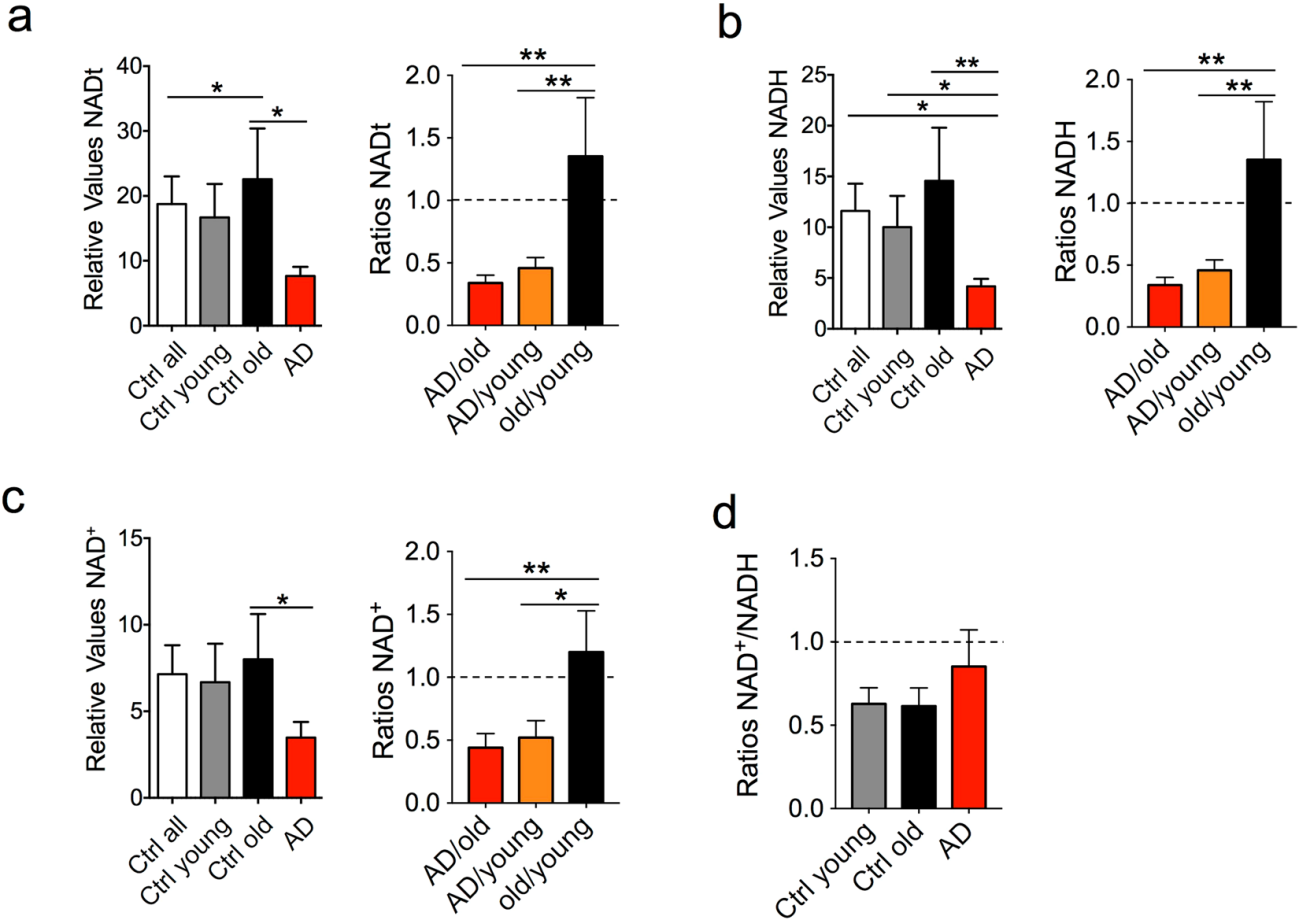
Bioenergetics parameters related to mitochondrial respiration. Nicotinamide metabolites, including NADt (**a**), NADH (**b**), and NAD^+^ (**c**) were decreased in LOAD samples compared to young and old controls, while the RRs (ratios NAD^+^/NADH) were not different between controls, but slightly increased in LOAD fibroblasts compared to all, young, and old control cells (**d**). Shown are relative values and ratios comparing AD with old and young and old with young samples. Two experiments were performed in *n* = 3 replicates. *P < 0.1; **P < 0.05; ***P < 0.01 (detailed statistical data are provided in Supplementary Table 2).

**Figure 4:**
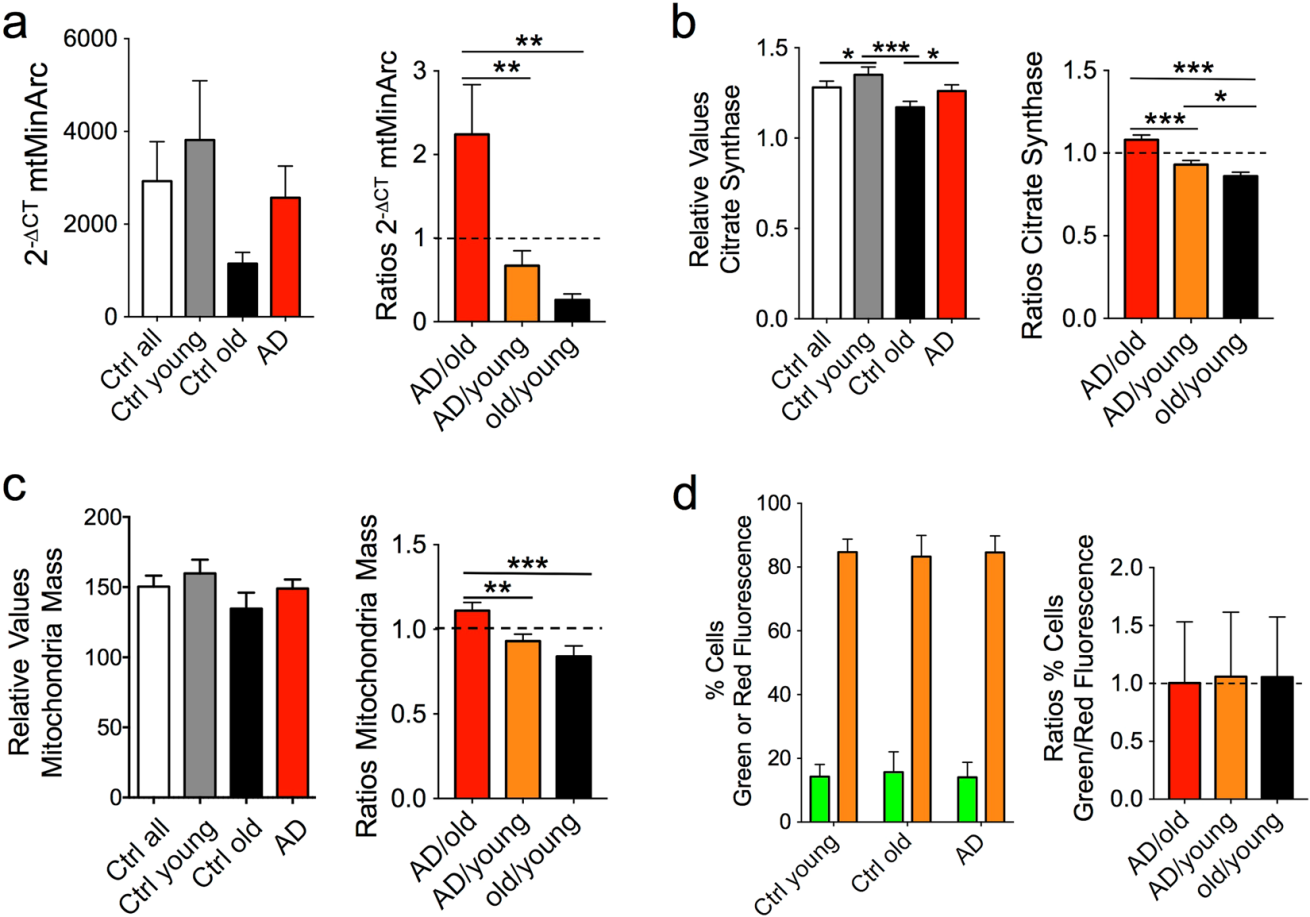
Parameters related to mitochondria mass and membrane integrity. qPCR experiments amplifying a region of the mtMinArc (**a**), CS (**b**), and MitoTracker assays (**c**) showed reduction of mitochondrial DNA amplification, CS activity, and mitochondria staining in healthy old and LOAD cells, respectively, versus young controls. FCM in combination with the MitoPT^®^ dye JC-1 showed no significant difference in the ratios of red and green fluorescent cells between control and LOAD samples indicating no loss of transmembrane electrical potential gradient (Δψ_m_) (**d**). The assay measures confirmation changes of the positively charged dye according to its localization in the cell, i.e., green fluorescence when accumulating in the cytoplasm and orange/red fluorescence when distributing to mitochondria. Orange/red fluorescence is indicative of dye accumulation in mitochondria due to an intact Δψ_m_, while green fluorescence indicates loss of Δψ_m_ due to mitochondrial damage of the ETC, and proton and pH gradient. Shown are relative values and ratios comparing AD with old and young and old with young samples. Two experiments were performed in *n* = 3 replicates. *P < 0.1; **P < 0.05; ***P < 0.01 (detailed statistical data are provided in Supplementary Table 2).

To assess the fibroblasts’ respiratory capacities, we investigated several parameters related to mitochondrial function and integrity. The mitochondrial redox potential was determined through the levels of total nicotinamide adenine dinucleotide (NADt), reduced NADH, and oxidized NAD^+^. There was a reduction of all NAD metabolites in LOAD cells compared to young and old controls, but not in old compared to young controls (Fig. 3a–c). In addition, the calculated redox ratios (RR), which indirectly measure the relative amounts of oxidized NAD^+^ to reduced NADH, were slightly increased in LOAD fibroblasts when compared to young and healthy old cells, while these ratios were not different in the latter two cell populations (Fig. 3d). Measured changes in mitochondrial function could be due to changes in mitochondrial mass. We therefore quantified mitochondria by determining DNA copy numbers using a PCR that targets mtMinArc^20^ (see methods for details), the activity of citrate synthase (CS)^21^, and quantification of mitochondria in MitoTracker assays. These assays showed that PCR amplification of mtMinArc DNA, CS activity and the relative numbers of mitochondria were decreased in healthy old and LOAD fibroblasts when compared to young controls, with more reduction in old than in LOAD cells (Fig. 4a–c). Together these data indicate that both old and LOAD fibroblasts have diminished mitochondria mass and that LOAD cells have a defective redox potential, which was disease-specific and not a function of age.

In LOAD fibroblasts, the increase in PPR and proton leak in the Seahorse experiments (Fig. 1b,c) are consistent with the observed dysbalance in the redox potential (Fig. 3) and could be indicative of an insufficiently functioning mitochondrial electron transfer chain (ETC) due to transmembrane instability, proton leakage, or loss of its electrical potential gradient (Δψ_m_). We, therefore, analyzed the redistribution of the MitoPT^®^ dye JC-1 across the mitochondrial inner cell membrane as an indicator of membrane damage and loss of Δψ_m_ by Flow Cytometry (FCM) (Supplementary Fig. 2). The results showed no significant differences in the ratios of green to red fluorescent cells between LOAD, healthy old, or young samples, indicating no loss of Δψ_m_ (Fig. 4d).

**Figure 5:**
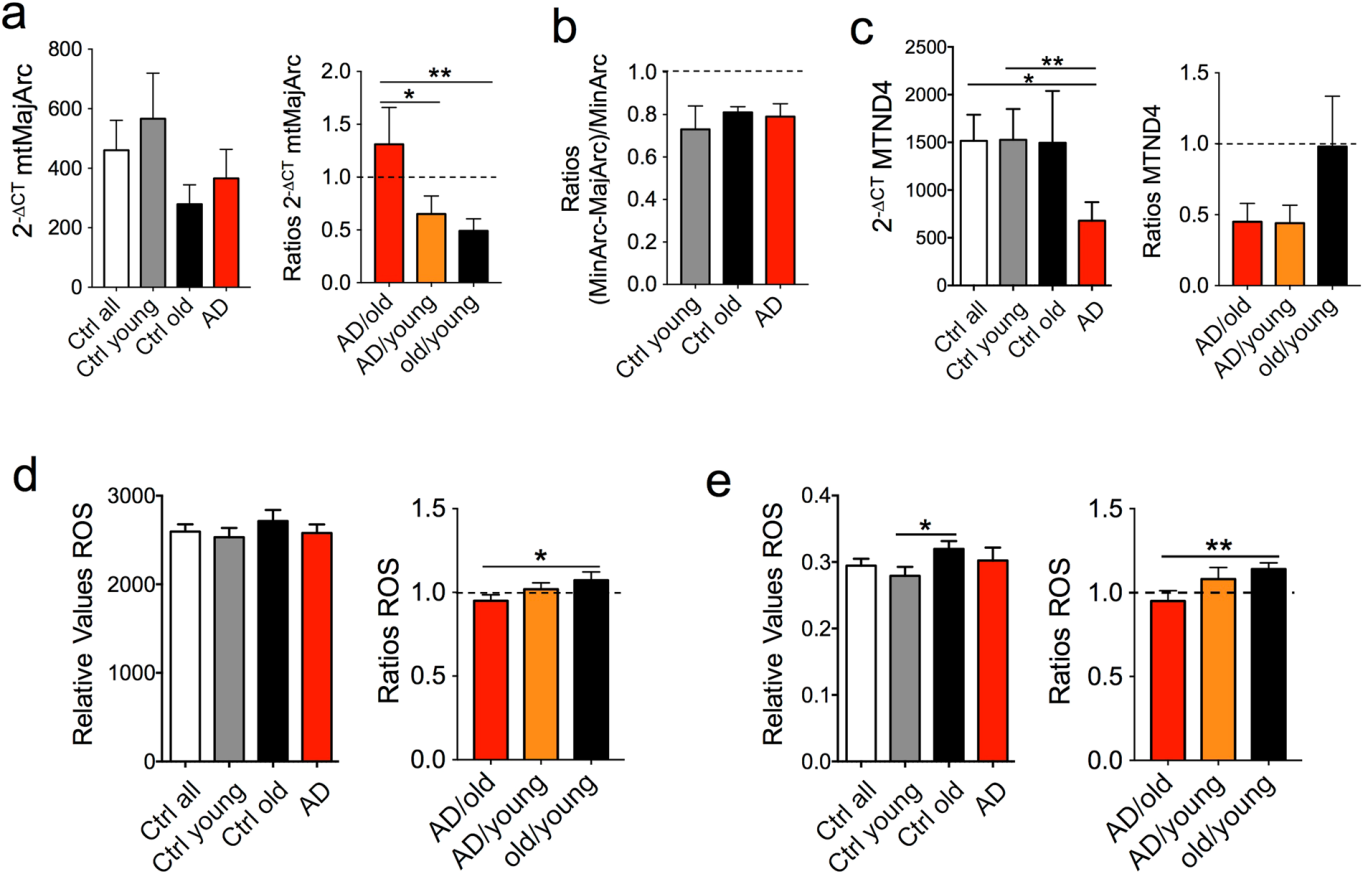
Parameters related to mitochondrial DNA integrity and transcription. qPCR experiments amplifying the 4977 “common deletion” within the mtMajArc showed reduced PCR amplification in old and LOAD cells (**a**), but no differences of calculated ratios over mtMinArc amplification (**b**). Expression of the MTND4 gene was decreased in LOAD cells when compared to all, young, and healthy old controls (**c**). ROS levels measured with the OxiSelect (**d**) or MitoSox (**e**) assays were slightly increased in LOAD and significantly increased in healthy old cells, compared to young controls. Shown are relative values and ratios comparing LOAD with old and young and old with young samples. Two experiments were performed in *n* = 3 replicates. *P < 0.1; **P < 0.05; ***P < 0.01 (detailed statistical data are provided in Supplementary Table 2).

In addition to mitochondrial mass, changes in mitochondrial activity could be due to mitochondrial DNA deletions. Such deletions and, in particular, a “common” 4977 bp deletion in the mtMajArc of the mitochondrial genome, are associated with oxidative stress in normal aging and in LOAD^22^. To assess if this deletion occurred in our fibroblast samples, we employed the same PCR strategy used for detecting mtMinArc, targeting a region of the mitochondrial NADH:Ubiquinone Oxidoreductase Core Subunit 4 (MTND4) gene in mtMajArc^20^. Although mtMajArc was less amplified in healthy old and LOAD cells than young controls (Fig. 5a), the ratios of mtMajArc over mtMinArc expressions were similar in all samples, indicating no increase in the 4977 deletion in healthy old or LOAD cells (Fig. 5b). When we assessed MTND4 expression, we found less expression in LOAD cells compared to young and old cells, suggesting diminished transcriptional activity of the MTND4 gene in LOAD (Fig. 5c). Finally, mitochondrial DNA is highly susceptible to the damaging effects of reactive oxygen species (ROS), which have been implicated in causing DNA deletions^22–24^. In two separate assays we detected slightly higher ROS levels in healthy old and LOAD cells when compared to young controls, which were significant in the old, but not in the LOAD samples (Fig. 5d,e). These data indicated that ROS production was not disease-specific and a function of age.

### Gene Expression Profiles Related to Glycolysis, NAD Metabolism, and CAC

**Figure 6:**
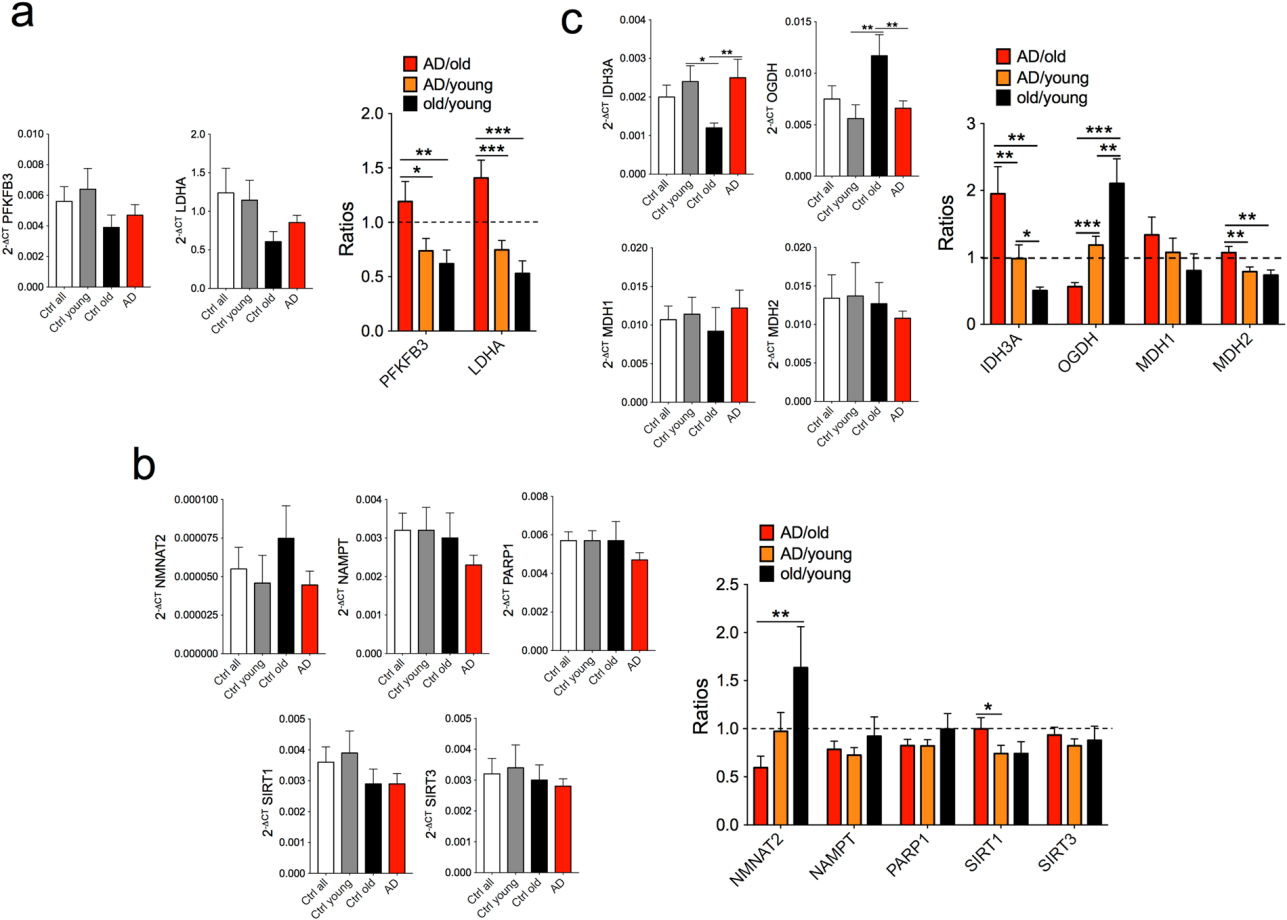
mRNA expression profiles of genes related to glycolysis, NAD metabolism, and CAC activity. (**a**) Gene expression profiles of PFKFB3 and LDHA showed downregulation in healthy old and LOAD cells, but a relative increase in the ratios of LOAD versus old fibroblasts when compared to LOAD or healthy old versus young cells. (**b**) Gene expression profiles of NMNAT2, NAMPT, PARP1, SIRT1, and SIRT3 demonstrated predominant downregulation in LOAD cells, including a significant decrease or increase of NMNAT2 in LOAD or old controls, respectively. (**c**) Gene expression of IDH3A, OGDH, MDH2, and cytosolic MDH1 showed significant differences between LOAD and old cells with increased expression of IDH3A, MDH1 and MDH2 in LOAD and decreased expression of IDH3A and MDH2 in old cells. In contrast, OGDH was decreased in LOAD and increased in old fibroblasts. Experiments were performed in *n* = 3 replicates. *P < 0.1; **P < 0.05; ***P < 0.01 (detailed statistical data are provided in Supplementary Table 2).

To gain insight into possible mechanisms of increased glycolysis and abnormal redox potential in LOAD cells, we analyzed the expression of key genes in the glycolytic pathway, NAD metabolism, and the citric acid cycle (CAC). First, we determined the expression of PFKFB3, which converts fructose-6-phosphate to fructose-2,6-bis-phosphate (F2,6BP), an allosteric activator of phospho-fructo-kinase (PFK), which is the rate-limiting enzyme in glycolysis, and lactate dehydrogenase A (LDHA), which converts pyruvate to lactate and NADH to NAD^+^, and vice versa. Both of these molecules have previously been associated with AD^25–27^. We found that expression of PFKFB3 and LDHA were decreased in old controls and LOAD cells when compared to young fibroblasts, but they were increased in LOAD compared to healthy old cells (Fig. 6a). We also determined the expression levels of Nicotinamide mononucleotide adenylyltransferase 2 (NMNAT2), Nicotinamide phosphoribosyltransferase (NAMPT), Sirtuin 1 and 3 (SIRT1, 3), and Poly [ADP-ribose] polymerase 1 (PARP1) (Fig. 6b). These molecules are involved in NAD metabolism, specifically in *de novo* synthesis or recycling and the hydrolysis of NAD^+^ to Nicotinamide (NAM), and they connect metabolic with other pathways in cellular function^28^. In addition, they have been implicated in aging and neurodegeneration, including AD^28–31^. While we observed no cohort differences in SIRT1 and 3, we found a slight downregulation of NAMPT and PARP1 in LOAD fibroblasts when compared to young and old control cells. Most strikingly, there was a significant difference of NMNAT2 expression between the ratios of LOAD over old controls and old over young controls, with downregulation in the former and upregulation in the latter. These data are consistent with our observed decrease of NAD metabolites in LOAD and an increase in healthy old cells (see Fig. 3), suggesting that the abnormal redox potential in LOAD may be in part due to a reduction in NAD synthesis and recycling.

Finally, we analyzed the expression of key genes in CAC that convert NAD^+^ to NADH, including Isocitrate dehydrogenase 3 (IDH3A), oxoglutarate dehydrogenase (OGDH), malate dehydrogenase 2 (MDH2), and its cytosolic isoform MDH1 (Fig. 6c). We found a significant upregulation of MDH2 and IDH3A in the ratios of LOAD over old or young controls, and a downregulation in old over young samples. Interestingly, the opposite was the case for OGDH, whose expression was significantly downregulated in LOAD and upregulated in old cells, comparing the ratios of LOAD with old and old with young fibroblasts. In addition, there was slightly more expression of MDH1 in LOAD cells compared to young and old controls. These data show that despite the diminished mitochondrial mass in both healthy old and LOAD cells, expression of CAC key enzymes involved in NAD^+^ reduction were increased, and this was more pronounced in LOAD fibroblasts.

### Correlation of Parameters in Bioenergetics and Metabolism with Age and Disease

**Figure 7:**
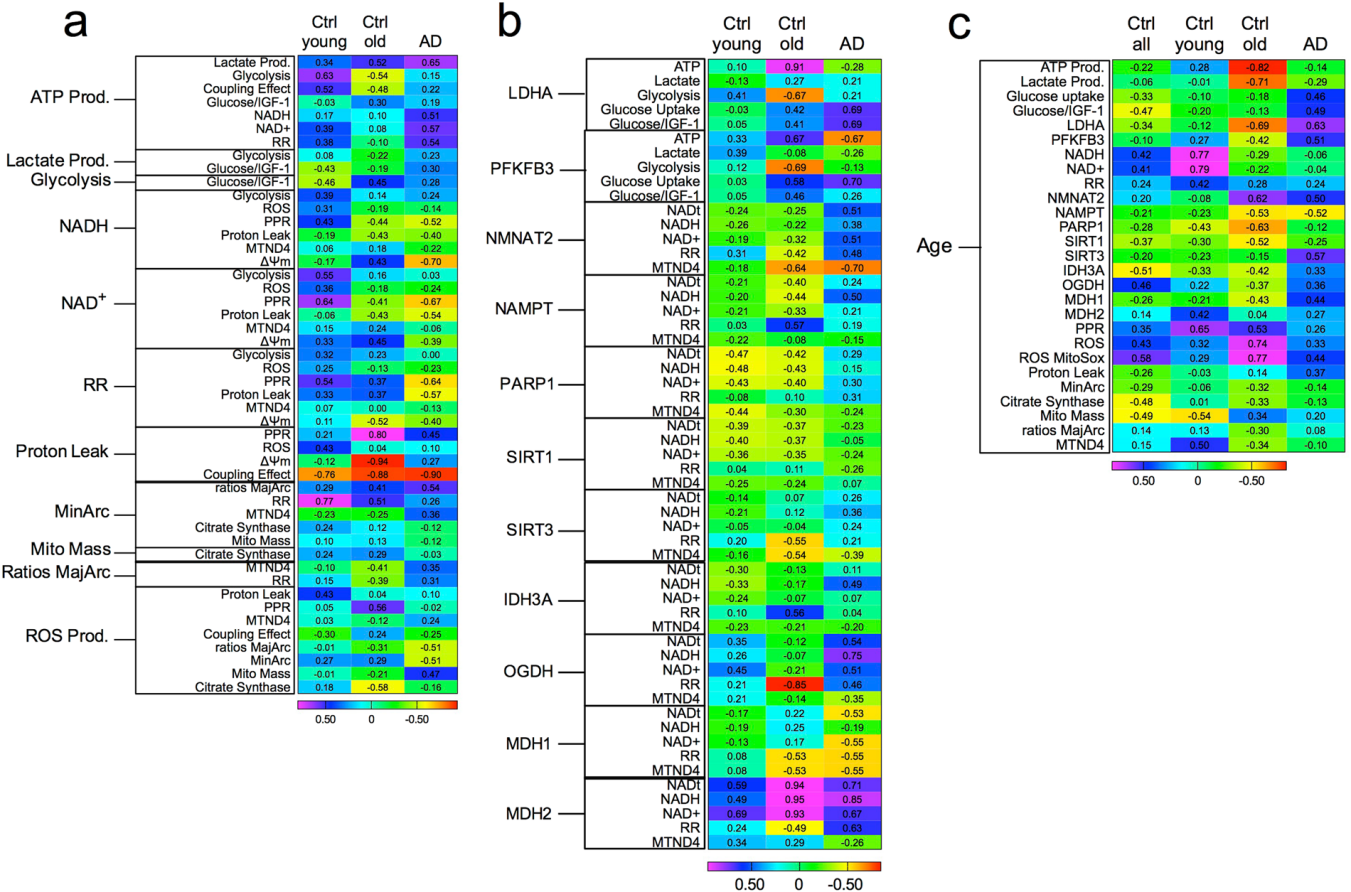
Heat maps of data correlations. Data related to energy production, mitochondrial function or integrity (**a**), and gene expression (**b**) were correlated within (**a**,**b**) or with age (**c**). Positive (blue) or negative (red) correlations (R values) are shown.

To determine the relationships of energy production, glycolysis, and mitochondrial function between LOAD, healthy old and young fibroblasts, we conducted a series of correlation analyses (Fig. 7a–c). The levels of overall energy production (ATP) strongly correlated positively with metabolic parameters in LOAD and young fibroblasts when compared to old cells, and were more strongly associated with glucose uptake and IGF-1 signaling in LOAD or healthy old than in young cells (Fig. 7a). As for the metabolites related to mitochondrial redox potential, they strongly correlated negatively with mitochondrial function and integrity in LOAD cells, consistent with an inefficiently functioning ETC and an abnormal RR. In addition, mitochondrial mass and DNA damage positively correlated with parameters of mitochondrial function and integrity, and negatively with ROS production in LOAD cells.

As for gene expression profiles, PFKFB3 and LDHA had strong positive correlations with glucose uptake in the absence or presence of IGF-1, but predominant negative correlations with glycolysis, ATP and lactate production in both LOAD and healthy old cells (Fig. 7b). For molecules related to NAD synthesis, recycling, and metabolism, the expression of NMNAT2, NAMPT, PARP1, and SIRT3, but not SIRT1, had strong positive correlations with NADt, NADH, NAD^+^, and RR levels in LOAD cells, while correlations in\ both old and young samples were negative (Fig. 7b), implying a compensatory increase in gene expression as a homeostatic attempt to sustain NAD metabolism in LOAD cells. Finally, the analysis of NAD metabolites with the expression of CAC enzymes demonstrated strong positive correlations with OGDH in young and LOAD, but not in healthy old cells (Fig. 7b). Strikingly, there were strong positive correlations with MDH2 expression in all samples, while expression of cytosolic MDH1 negatively correlated with the NAD metabolites in young and LOAD, and positively in healthy old cells. As for RR, its levels correlated strongly positively with IDH3A and negatively with OGDH, MDH1, and MDH2 in old cells, while both young and LOAD fibroblasts had positive correlations, except for MDH1, which also correlated negatively with RR in LOAD cells. Together, the predominant positive correlations of CAC enzyme expression with NAD metabolite or RR levels in LOAD cells could be a reflection of the increased RR seen in these cells.

Lastly, we compared the bioenergetics parameters and the results from the gene expression analyses with age (Fig. 7c). In all healthy control samples, overall energy production and glycolytic metabolites were diminished with age, while mitochondrial respiration increased, despite an increase in ROS production and DNA damage, and a reduction in mitochondrial mass. In comparison to young fibroblasts, overall energy production and the metabolites from glycolysis and mitochondrial respiration decrease with age in both healthy old and LOAD cells, despite an increase in the redox potential and PPR, and an increase in glucose uptake and IGF-1 signaling in LOAD cells. Consistently, expression levels of PFKFB3, LDHA, NMNAT2, and SIRT3 increased with age in LOAD, but decreased in young and old controls. As for the other NAD-related enzymes, NMNAT2, PARP1, and SIRT1 declined with age in all samples. Interestingly, we found an increase of the CAC enzymes IDH3A, OGDH, MDH1 with age in LOAD, but a decrease in old and young controls. Finally, a decrease in bioenergetic parameters related to mitochondrial mass, integrity and damage seemed to be more pronounced in old than in LOAD cells.

## Discussion

There are a number of evidence-based hypotheses as to the cause of AD. Aβ-containing plaques and tangles of p-tau have long been considered the “hallmarks” of AD pathology. However, there is increasing evidence that in LOAD the accumulation of these toxic molecules may not be the sole or initial cause of the disease and may instead be consequences of other causative factors^4–6,9,10^. LOAD is most likely characterized by a combination of several interacting pathological events that are closely related to changes with age and that include, amongst others, bioenergetic, metabolic, neurovascular, and inflammatory processes^5,6,10^. A key determinant of LOAD is aging and, in fact, many, if not all, pathological events in LOAD are also part of the normal aging process. For example, there is strong evidence that cellular energy metabolism changes with age, and that bioenergetic anomalies may trigger processes, such as resistance to growth factor/insulin signaling, neuroinflammation, cell membrane changes, and microvascular disease, which all contribute to senescence and age-related neurodegeneration, including LOAD^7,8,10^.

Bioenergetics consists of a multitude of different cellular pathways and processes that include mitochondrial respiration and the metabolism of various fuel molecules and their downstream products to produce and utilize energy. A major energy substrate, especially in brain, is glucose, which is metabolized via cytosolic aerobic glycolysis to pyruvate or under low oxygen conditions via anaerobic glycolysis to lactate. Under usual oxygen levels, pyruvate is processed in the mitochondria through CAC and OxPhos to generate ATP. In addition, glycolysis oxidizes the reduced form of NADH to NAD^+^, which is an important oxidizing agent involved in redox reactions and electron transfer. In the mitochondria, OxPhos is coupled to CAC and can metabolize substrates from different sources, including carbohydrates (i.e., pyruvate or lactate produced from glycolysis), ketones, fatty acids, glutamine, and others. Contrary to glycolysis, OxPhos largely results in the reduction of NAD^+^ to NADH. Mitochondrial energy production depends on oxygen and is highly efficient, producing about 34–36 mol of ATP from one mol glucose with CO_2_ and H_2_O as waste products. In contrast, both forms of glycolysis generate ATP at low efficiency (about two mol per mol of glucose). However, under most conditions aerobic glycolysis can produce ATP at a faster rate than OxPhos, and glycolysis is usually increased in growing or stressed cells. A third mechanism to metabolize glucose is through PPP, in which glucose-6-phosphate is converted to ribose-5- and xylulose-5-phosphate leading to the reduction of NADP^+^ to NADPH, which pathway is used for fatty acid biosynthesis and regeneration of reduced glutathione.

A key bioenergetic factor that has long been suspected in senescence and neurodegeneration is oxidative stress, which produces DNA-damaging ROS^22,24^, and elevated ROS is an early event in AD^32^. For example, oxidative stress has been implicated in damaging the brain’s vascular endothelium in early stages of AD, leading to microvascular disease and blood brain barrier dysfunction as significant contributors to LOAD pathology^33,34^. Oxidative stress is particularly harmful in neurons, because these cells almost entirely rely on OxPhos for energy production, due to a paucity of key enzymes in the glycolytic pathway, including PFKFB3, which is highly degraded at the UPS due to high activity of the E3 ubiquitin ligase, anaphase-promoting complex/cyclosome (APC/C)-Cdh1, in neurons^12,13^. Therefore, neurons can’t efficiently switch from OxPhos to glycolysis (Warburg effect) to meet energy demands, making them especially vulnerable to stress and bioenergetic changes. Mitochondrial damage in neurons leads to reduced efficiency and a compensatory increase in OxPhos (inverse Warburg effect), and the cells may also abnormally upregulate glycolysis^14–18^. This could be a consequence of numerous factors, such as mitochondrial dysfunction, possibly through age-associated mitochondrial DNA deletions^35,36^, resistance to growth factor and IGF-1/insulin signaling^10,19^, and risk factors such as Apoε4, which has been implicated in damaging the oxidative system^37–39^. In addition, astrocytes increase metabolic rates through upregulation of glycolysis (Warburg effect) to produce lactate and support the increased energy demand in neurons^14–18^, which may lead to intracellular molecular changes and astrogliosis, further contributing to AD pathology^40–42^.

**Figure 8:**
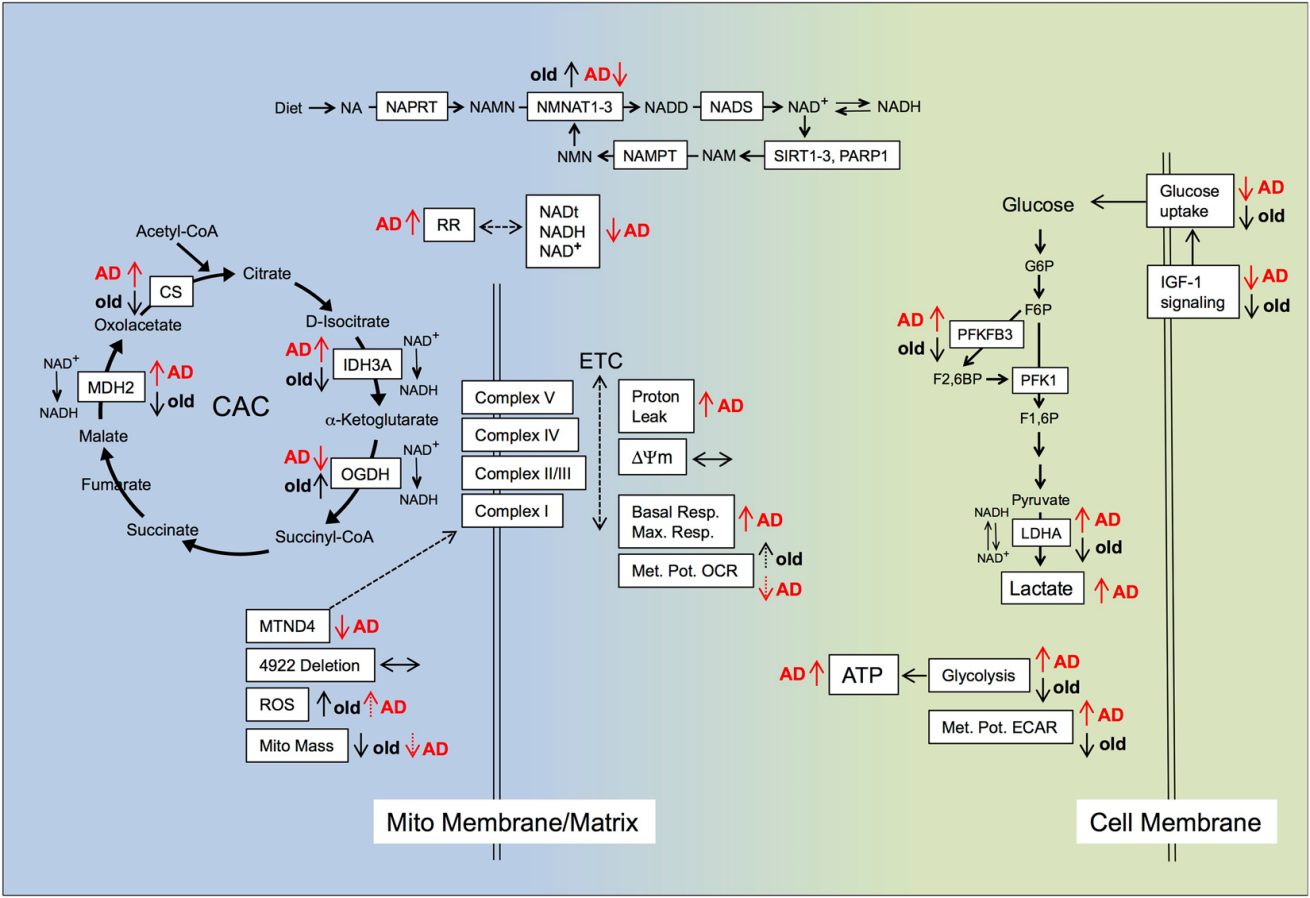
Schematic summary of bioenergetic changes in LOAD and old control fibroblasts. Parameters related to mitochondrial function, mass, integrity, OxPhos, and NAD metabolism are in the blue area and those related to glycolysis in the green area. Arrows indicate up-regulation, down-regulation, or no changes in parameters for LOAD relative to young or old controls, and old relative to young controls. While alterations in glucose uptake, IGF-1 signaling, mitochondrial mass, ROS production, DNA deletion, and membrane stability follow similar patterns in healthy old and LOAD cells, changes in glycolysis, NAD metabolism, CAC activity, expression of MTND4, proton leak, and respiratory potential appear to be more specific to either LOAD or aging. See text for details. Additional abbreviations: G6P: Glucose-6-phosphate; F6P: Fructose-6-phosphate; F1,6 P: Fructose-1,6-phosphate; F2,6BP: Fructose-2,6-bis-phosphate; NA: nicotinic acid; NAMN: nicotinate mononucleotide; NADD: nicotinic acid adenine dinucleotide; NAM: Nicotinamide; NMN: nicotinamide mononucleotide; NAPRT: Nicotinate Phosphoribosyltransferase; NADS: NAD synthetase.

The above conceptual outline of a role for dysfunctional bioenergetics in LOAD has not yet been experimentally proven. Studying bioenergetics in human aging and neurodegeneration is difficult, as the availability of relevant human material is limited and experimental systems are often hard to interpret due to high inter-subject variability, insufficiencies of model systems that mimic the human condition, species-specific biology, and other complicating factors, including limits of technology for studying live brains. The goal of our study was to determine if bioenergetic changes occur in cells from LOAD patients and how changes may relate to normal aging. Age related changes occur throughout the body, and factors underlying LOAD may be studied in brain as well as peripheral cells, including primary skin fibroblasts, which have been used in many studies as model system to investigate cell functions in context of genetic predisposition, e.g., familial forms of AD, cellular toxins, therapeutic drugs, or as a diagnostic tool. However, to our knowledge, there has been no published investigation that comprehensively assessed and compared the bioenergetics profiles in unmodified naïve LOAD and healthy control fibroblasts, as a function of age and disease. Our data reveal that LOAD cells have an overall increase in basal and respiratory consumption, but an impairment of their mitochondrial metabolic potential, which was associated with a decrease in NAD metabolites and altered CAC activity, but not with a disease-specific reduction of mitochondria mass, transmembrane instability, ROS production, or mitochondrial DNA deletions. The observed changes in mitochondrial function in LOAD cells could be related to altered gene expression profiles, including decreased expression of MTND4 and key molecules involved in NAD synthesis, recycling, or metabolism, and upregulation of enzymes in the CAC. In addition, LOAD cells shift their energy production to glycolysis resulting in augmented ATP and lactate production. The increase in glycolytic activity occurs in spite of an inability to augment glucose uptake in response to IGF-1. Heightened glycolysis in combination with a decreased mitochondrial metabolic potential and a defective redox potential in LOAD fibroblasts appears to be disease- and not age-specific features, while the inability to increase glucose uptake in response to IGF-1 signaling is a characteristic of both healthy old and LOAD cells. A schematic summary of these results is provided in Fig. 8.

These findings have several implications for understanding the role of bioenergetic changes in LOAD pathology and aging. First, the increase of glycolysis with an upregulation of glycolytic enzymes and augmented ATP and lactate production, together with an age-associated increase in glucose uptake, IGF-1 signaling, and PFKFB3 and LDHA gene expression in LOAD cells could be a compensatory mechanism to meet heightened substrate demand in the face of failing mitochondrial ability to sustain overall energy production, as indicated by their diminished mitochondrial metabolic potential and a reduction in NAD metabolism. The increase of glycolysis, however, is not associated with an overall increase in glucose supply, because, as in fibroblasts from healthy older subjects, overall glucose uptake and the response to IGF-1 signaling are impaired in LOAD cells. These data support the hypothesis that resistance to IGF-1/insulin-signaling is associated with age per se, as it is seen in both aging and AD pathology^10,19^, and also suggest that in LOAD upregulation of glycolysis may be an independent mechanism that is unrelated to insulin/IGF-1 resistance. Second, the impaired dynamic mitochondrial metabolic potential and the overall increase in basal and oxidative consumption in LOAD cells could be an expression of decreased NAD metabolism, increased CAC activity, and an abnormally elevated RR - the redox potential - which reflects the cellular reducing potential and whose disturbance can be an indicator of oxidative stress or imbalance. Specifically, the reduction of tNAD, NAD^+^ and NADH could be a consequence of the observed decrease in the expression of key molecules in NAD synthesis, recycling, or metabolism, and, in particular NMNAT2, which acts on converting both *de novo* synthesized nicotinate mononucleotide (NAMN) and recycled nicotinamide mononucleotide (NMN) to nicotinic acid adenine dinucleotide (NADD) (see Fig. 8). In addition, other parameters likely contribute to a disturbed mitochondrial metabolic and redox potential, including the diminished expression of MTND4, a defective ETC, as suggested by the increase in proton leak and PPR, and the observed increase in CS activity relative to old cells, and alterations in the expression of the CAC molecules IDH3A, OGDH, and MDH2 that are involved in reducing NAD^+^ to NADH. Finally, as the ratio of the redox pair NAD^+^/NADH is tightly linked to both the activity of the glycolytic pathway and OxPhos, the relative increase in the RR could be a consequence of the observed altered prominence of these processes, and of damaged mitochondria. There is evidence that NAD^+^ inhibits the production of and protects against the damaging effects of ROS^43,44^, which have been implicated in AD pathogenesis^22,24^. Overall ROS levels were slightly elevated in both healthy old or LOAD cells, but there was no increase in the 4977 “common” DNA deletion, indicating that changes in ROS are age-associated, and mitochondrial DNA damage through ROS is not a major factor in either aging or LOAD fibroblasts. Third, in terms of overall energy (ATP) production, LOAD fibroblasts were more closely related to young than old control cells, in particular, as indicated by their heightened glycolytic activity. In contrast, mitochondrial mass and their overall integrity in LOAD seemed to reflect more the profiles of those in old cells. A key difference between healthy old and LOAD cells, however, appears to be a defective ETC and RR, and dysbalances in the synthesis, recycling, and use of NAD metabolites, their utilization in the CAC, and their correlations to the bioenergetic profiles in general, as well as to their age-associations. This unique metabolic profile of LOAD fibroblasts may be the reason for their less efficiently functioning respiration and their compensatory upregulation of glycolysis.

Taken together, our data are partially consistent with an earlier report documenting increased glycolysis and a decrease in oxidative metabolism in skin fibroblasts from AD patients^45^. They are also consistent with several cellular features that have been associated with LOAD in general, including oxidative stress, diminished mitochondrial potential, defective or reduced mitochondria, and IGF-1/insulin resistance, but don’t support other characteristics, such as mitochondrial DNA deletion and increased ROS production (reviewed in^5–7,10,46^). It should be kept in mind that skin fibroblasts are not the primary cell type that is affected in LOAD and that changes in cell function are most likely partly cell-specific. In addition, as discussed above, the pathogenesis of LOAD is probably multifactorial with bioenergetics being one part of risk determination. Although our study does not provide a causative mechanism on how these metabolic changes arise or interact to drive disease pathogenesis, the current results suggest potential targets to study. Specifically, our data reveal that LOAD fibroblasts appear to exhibit a Warburg-type effect that is disease-specific and unrelated to age. Although it needs to be determined if this feature also applies to AD-relevant cell types, such as neurons and astrocytes, our findings could support the “Warburg/inverse Warburg” hypothesis, in that brain cells in LOAD may upregulate OxPhos and glycolysis due to a reduction of the mitochondrial metabolic potential, damage, or death, resulting in bioenergetic dysbalances. While upregulation of glycolysis may be beneficial in astrocytes to sustain heightened energy production, it may be detrimental in neurons, because downregulation of glycolytic activity through APC/C-Cdh1 mediated regulation of PFKFB3 levels protects these cells against oxidation, and upregulation of the glycolytic pathway causes oxidative stress and apoptosis^13^. In addition, while PFKFB3 promotes cell cycle and is anti-apoptotic in proliferating cells^47^, such induction of the cell cycle machinery in postmitotic neurons would have the opposite effect leading to apoptotic cell death. Finally, bioenergetics dysbalances may be inherent characteristics in LOAD, and in combination with other factors may contribute to its pathology. And, these defects may be system wide, due to underlying genomic and environmental factors, but have more evident consequences in brain, with its high energy-requirements.

## Methods

### Subjects and Cell Lines

Subjects (Supplementary Table 1) were recruited at the McLean Hospital Memory Diagnostic Clinic and diagnosed by a geriatric psychiatrist using the Diagnostic and Statistical Manual of Mental Disorders (DSM-IV) criteria and the Montreal Cognitive Assessment (MOCA) score. Fibroblasts were derived from skin biopsies or purchased from ATCC (D551 and HFF1 cell lines) and cultured in MEM media (Thermo Fisher Scientific, Waltham, MA) supplemented with 15% FBS, 100 U/ml Penicillin/Streptomycin (Thermo Fisher Scientific) and 2 mM L-glutamine (Sigma, St Louis, MO), and stored in the cell bank of the Program for Neuropsychiatric Research at McLean Hospital. All experiments and procedures were performed in accordance with relevant guidelines and regulations and were approved by the Partners Health Care Institutional Review Board. All subjects gave written informed consent.

### Seahorse XFp Cell Mito Stress Test

Seahorse XFp Cell Mito Stress Tests (Seahorse, Agilent Technologies, Santa Clara, CA) (Supplementary Fig. 1) were performed according to the manufacturer’s protocol on an XFp instrument. Two days prior assay, Seahorse culture plates were coated with 16.7 µl/ml Matrigel in DMEM/F12 media (Corning, Corning, NY) for 2 hrs at 37 °C and stored at 4 °C. One day prior assay, 10,000 fibroblasts per well were plated and cultured in MEM15 media overnight. At day of assay, XF assay medium was supplemented with 10 mM glucose, 1 mM pyruvate, and 2 mM glutamine, and the pH adjusted to 7.4. After assay performance, cells were stained with CyQuant solution (Life Technologies - Thermo Fisher Scientific, Carlsbad, CA) diluted in XF assay medium and incubated for 1 hr in 37 °C. Green fluorescence (excitation: 485/20, emission: 528/20) was measured using a Synergy HT BioTek plate reader (BioTek Instruments, Winooski, VT) and values used for data normalization. Data analysis was performed using the Seahorse XF^e^ Wave software, including the Seahorse XF Cell Energy Phenotype Test Report Generator.

### Assays for Detecting Metabolites

To detect metabolites, commercially available kits were used according to protocols provided by the manufacturers with modifications to determine optimal conditions and normalize data: *ATP Luminescence Detection Kit* (Abcam, #ab113849, Cambridge, UK) in combination with *CyQuant DNA stain* (Thermo Fisher Scientific); *NAD/NADH Assay Kit* (Abcam, #ab45348) in combination with *DC Protein Assay* (BioRad, Hercules, CA); *L-Lactate Detection Kit* (Abcam, #ab65331) in combination with *CyQuant DNA stain* (Thermo Fisher Scientific); *Citrate Synthase Activity Kit* (Abcam, #ab119692) in combination with *DC Protein Assay* (BioRad); *Glucose Uptake Cell-Based Assay* (Cayman, #600470, Ann Arbor, MI) in combination with *DC Protein Assay* (BioRad) and 50 ng/ml IGF-1 (Thermo Fisher Scientific)^48,49^; *OxiSelect Intracellular ROS Assay Kit (Green Fluorescence)* (Cell Biolabs, #STA-342, San Diego, CA) in combination with *Hoechst 33342* staining (Thermo Fisher Scientific). *MitoSox* and *MitoTracker assay kits* (Thermo Fisher Scientific, #M36008 and #M7514, respectively). Both MitoSox (red fluorescence) and MitoTracker (green fluorescence) staining and measurements were performed simultaneously in combination with *DC Protein Assay* (BioRad). Protein-normalized MitoSox data were normalized to protein-normalized MitoTracker results (see Supplementary Fig. 3). All assays were performed in 96 well formats or from cell lysates, and luminescence, fluorescence or absorbance measured on a Synergy HT BioTek plate reader (BioTek Instruments). Assays were performed twice and cell numbers ranged from 12,000 to 25,000 cells per 96 well cultures (ATP, glucose uptake, ROS, MitoSox/MitoTracker), or 100,000 to 500,000 cells pelleted for staining or cell lysis (citrate synthase, L-Lactate, Mito-PT FCM, NAD/NADH).

### MitoPT JC-1 Assay

The mitoPT JC-1 assay (ImmunoChemistry Technologies, Bloomington, MN) using the slow, lipophilic, cationic fluorescent dye 5,5′,6,6′-tetrachloro-1,1′,3,3′ tetraethylbenzimidazolocarbocyanine iodide (JC-1)^50^ was performed in combination with FCM on a BD Accuri C6 Flow Cytometer (BD Biosciences, San Jose, CA) according to protocols provided by the manufacturer. The gating strategy and the use of controls are presented in Supplementary Fig. 2.

### Preparation of Nucleic Acids and Polymerase Chain Reaction (PCR)

Cells were harvested, placed in 0.5 ml TRIzol Reagent (Thermo Fisher Scientific) and stored at −80 °C. 100 µl chloroform was added, the samples centrifuged, and the RNA-containing aqueous phase collected, mixed with an equal amount of 75% ethanol and RNA was purified using the RNeasy Mini Kit (Qiagen, Valencia, CA) including DNase digestion with RNase-free DNase I (Quiagen) for 15 min. DNA was extracted from the same samples by adding 150 µl 100% ethanol to the organic interphase, incubation at room temperature (RT) for 3 min, and centrifugation. The phenol/ethanol supernatant was removed and the DNA washed in 0.5 ml 0.1 M sodium citrate solution, incubated for 30 min at RT and centrifuged. The DNA was resuspended in 0.5 ml 75% ethanol, incubated for 10 min at RT, centrifuged, dried for 5 min, and dissolved in 8 mM NaOH.

Quantitative (q)PCR was performed with primers amplifying a region in the genomic mitochondrial minor arc (mtMinArc) or targeting the MTND4 gene located in the major arc (mtMajArc) together with primers targeting a region within the single-copy human genomic β2-microglobulin gene (B2M) as described^20^. PCR was conducted on a BioRad CFX Connect PCR cycler (BioRad) using probe-based multiplex TaqMan qPCR assays (Applied Biosystems, Waltham, Massachusetts) labeling the mtMinArc and mtMajArc probes with FAM, and the human B2M gene probe with VIC. 50 ng of template DNA was used in 20 μl reactions containing 10 μl TaqMan Universal PCR Master Mix, 50 nM mtMin/Arc or mtMaj/Arc primers together with 500 nM B2M primers and 250 nM TaqMan Probes (Applied Biosystems). The settings for the cycler were as follows: 10 min 95 °C, followed by 50 repeats of 15 sec 95 °C, 15 sec 55 °C, 1 min 60 °C. For detecting mRNA transcripts, 2 µg of purified RNA was treated with 2 µl DNAse I buffer and 1 µl rDNAse I (Ambion, Carlsbad, CA), incubated at 37 °C for 30 min, resuspended in 2 µl DNAse Inactivation Reagent, incubated at RT for 2 min, and centrifuged. The RNA-containing supernatants were collected and assessed for remaining mitochondrial DNA contamination in 20 µl PCR reactions containing 50 nM mtMinArc primers, iTaq Universal SYBR Green Supermix (BioRad) using the cycler settings as described above. For qPCR, 1 to 2 µg of total RNA was converted to cDNA using the High Capacity cDNA Reverse Transcription (Applied Biosystems) in 20 µl per reactions according to the protocol provided by the manufacturer. cDNA was then diluted 1:10 and qPCR performed with 50 nM published primers (see Supplementary Material) in PCR reactions using Taq Universal SYBR Green Supermix, and the following settings: genomic PCR according to^20^ using 50 cycles, and cDNA PCR at 95 °C/15 sec followed by 50 cycles at 95 °C/15 sec, 60 °C/1 min, and 72 °C/30 sec. PCR data were analyzed using the 2^−^Δ^CT^ method^51^ to normalize mitochondrial genomic mtMinArc and mtMajArc, and MTND4 against B2M, and the other transcripts against β-Actin (BACT). To calculate relative levels of mtMajArc DNA normalized values were used in the following formula: (mtMinArc-mtMajArc)/mtMinArc.

### Statistical analysis

Data were plotted as mean +/− standard error of the mean (SEM) from 2 independent experiments performed in triplicates (*n* = 3), unless otherwise stated. One-way analysis of variance (ANOVA) tests for independent measures were performed using the Social Science Statistics software (http://www.socscistatistics.com/Default.aspx). Differences of comparison were considered statistically significant when *P*-values were less than 0.05 (*P* < 0.05), while *P*-values between 0.05 and 0.1 were considered trend data. Detailed statistical data can be found in Supplementary Table 2.

### Data availability statement

All data generated or analyzed during this study are included in this published article (and its Supplementary Information files).

## Electronic supplementary material

Supplementary information
