# Supplementary material for: Late-onset Alzheimer’s disease is associated with inherent changes in bioenergetics profiles
Source: Sci Rep. 2017 Oct 25;7:14038. doi: 10.1038/s41598-017-14420-x (PMC5656579; doi:10.1038/s41598-017-14420-x)
Supplement: Supplementary file 1 — Supplementary information [file 41598_2017_14420_MOESM1_ESM.pdf]

Late-onset Alzheimer's disease is associated with inherent changes in bioenergetics profiles

Kai-C. Sonntag[1,2,3]*, Woo-In Ryu[1,2,3], Kristopher M. Amirault[1,2,3], Ryan A. Healy[1,2,3], Arthur J. Siegel[4], Donna L. McPhie[1,3], Brent Forester[1,5], Bruce M. Cohen[1,3]

[1]Department of Psychiatry, [2]Basic Neuroscience Division, [3]Program for Neuropsychiatric Research, [4]Internal Medicine Department, [5]Mood Disorders Division and Geriatric Psychiatry Research Program, McLean Hospital, Harvard Medical School, Belmont, Massachusetts 02478

*Correspondence to:
Kai-Christian Sonntag, M.D., Ph.D.,
Department of Psychiatry, Basic Neuroscience Division,
McLean Hospital, Harvard Medical School,
MRC 331, 115 Mill Street,
Belmont, MA 02478,
Phone: 617-855-3138,
Fax: 617-855-3793,
e.mail: ksonntag@mclean.harvard.edu

Supplementary Material

Supplementary Methods

Oligonucleotide primers for qRT-PCR. Primer sequences for qRT-PCR were deducted from the literature as follows: MTND4 [1]; B2M [2]; BACT [3]; LDHA [3]; PFKFB3 [4]; NMNAT2 [5]; NAMPT [6]; PARP1 [7]; SIRT1 [8]; SIRT3 [8]; MDH1 [9]; MDH2 [9]; OGDH [10]; IDH3A: FW 5'-TGCTGCCAAAGCACCTATTCA-3', RV 5'-CCCGGTCTGCCACAAAGT-3' (derived from PrimerBank: https://pga.mgh.harvard.edu/primerbank/).

Supplementary Tables and Figures

sample #	age	Sex	Diagnosis
367	21	M	Ctrl
294	23	M	Ctrl
460	25	M	Ctrl
458	26	M	Ctrl
359	31	M	Ctrl
298	33	M	Ctrl
366	35	M	Ctrl
152	40	M	Ctrl
190	42	M	Ctrl
226	45	M	Ctrl
135	49	M	Ctrl
365	52	M	Ctrl
316	54	M	Ctrl
**ave young**	**36.6**		
14	55	M	Ctrl
342	57	M	Ctrl
491	61	M	Ctrl
127	62	M	Ctrl
357	65	M	Ctrl
205	67	M	Ctrl
405	75	F	Ctrl
**ave old**	**63.1**		
330	56	M	AD
374	59	F	AD
231	65	M	AD
311	70	M	AD
369	76	M	AD
281	79	M	AD
402	70	F	AD
399	71	F	AD
404	81	M	AD
332	82	F	AD
**ave AD**	**70.9**		

Supplementary Table 1 | Summary of fibroblasts samples used in this study.

Figure 1

Values	all/old			all/AD			young/old			young/AD			old/AD			all groups		
	p-value	*F*		*p*-value	*F*		*p*-value	*F*		*p*-value	*F*		*p*-value	*F*		*p*-value	*F*	
Fig. 1b Spare Resp. Cap.	0.471589	1,24	0.53501	**0.106101**	1,27	2.79516	0.27863	1,17	1.25256	**0.062741**	1,20	3.88421	0.533765	1,15	0.40569	0.209233	3,44	1.57365
Fig. 1b Glycol. Cap.	0.306941	1,24	1.08969	**0.096018**	1,27	2.9745	0.128479	1,17	2.55337	0.349118	1,20	0.91926	0.019566	1,15	6.83033	0.119992	3,44	2.05491
Fig. 1b PPR	0.948499	1,24	0.00426	**0.106976**	1,27	2.81568	0.925229	1,17	0.00907	0.1335	1,20	2.44613	0.240079	1,15	1.49652	0.350623	3,44	1.12162
Fig. 1d baseline OCR	0.509416	1,24	0.44855	0.294311	1,27	1014384	0.357664	1,17	0.89392	0.56613	1,20	34038	0.197269	1,15	1.8205	0.523812	3,44	0.75783
Fig. 1d stressed OCR	0.850808	1,24	0.03615	0.184284	1,27	1.85653	0.788594	1,17	0.0742	0.293511	1,20	1.16384	0.281439	1,15	1.24838	0.480354	3,44	0.83797
Fig. 1d baseline ECAR	0.672606	1,24	0.18302	0.210291	1,27	1.64687	0.507377	1,17	0.45863	0.162704	1,20	2.10099	0.590647	1,15	0.30212	0.468226	3,44	0.86141
Fig. 1d stressed ECAR	0.758181	1,24	0.09697	0.166937	1,27	2.01754	0.640263	1,17	0.2264	0.264006	1,20	1.32081	0.22657	1,15	1.59012	0.449259	3,44	0.89914
Fig. 1d met. pot. OCR	0.470548	1,24	0.53756	0.871455	1,27	0.02669	0.266285	1,17	1.32124	0.468956	1,20	0.54497	0.564773	1,15	0.34666	0.695178	3,44	0.48391
Fig. 1d Met. Pot. ECAR	0.109992	1,24	2.75453	0.95892	1,27	0.0027	**0.024352**	1,17	6.10551	0.458452	1,20	0.57156	**0.024352**	1,15	6.10551	0.205508	3,44	1.58924

Ratios	AD/old - AD/young			AD/old - old/young			AD/young - old/young			all groups		
	p-value	*F*		*p*-value	*F*		*p*-value	*F*		*p*-value	*F*	
Fig. 1c Basal Resp.	**0.000138**	1,18	23.20985	**0.000548**	1,15	19.10171	1.31773	1,15	0.268978	**0.000074**	2,24	14.51661
Fig. 1c ATP Prod.	**0.000194**	1,18	21.72551	**0.00078**	1,15	17.6025	1.05738	1,15	0.320112	**0.000121**	2,24	13.43865
Fig. 1c Proton Leak	**0.008583**	1,18	8.69725	**0.008583**	1,15	8.69725	1.58119	1,15	0.227815	**0.002196**	2,24	7.98551
Fig. 1c Max. Resp.	**0.103143**	1,18	2.94797	**6.01451**	1,15	0.026912	0.308253	1,15	1011241	**0.045822**	2,24	3.51525
Fig. 1c Spare Resp. Cap.	0.39868	1,18	0.74739	0.927303	1,15	0.00861	0.533765	1,15	0.40569	0.658546	2,24	0.42508
Fig. 1c Non-Mito Resp.	0.603252	1,18	0.27987	0.183835	1,15	1.94127	0.339917	1,15	0.97159	0.411797	2,24	0.92085
Fig. 1c Coupling Effect	**<0.00001**	1,18	129.0867	**<0.00001**	1,15	53.60973	0.305148	1,15	1.1273	**<0.00001**	2,24	58.32071
Fig. 1c Glycol. Cap.	**0.001303**	1,18	14.46224	**0.000114**	1,15	26.71374	**0.019566**	1,15	6.83033	0.000023	2,24	17.21279
Fig. 1c PPR	0.144624	1,18	0.32579	**0.026623**	1,15	6.04141	0.240079	1,15	1.49652	**0.051876**	2,24	3.35562
Fig. 1d Baseline OCR	0.380595	1,18	0.80795	**0.05041**	1,15	4.2467	0.197269	1,15	1.8205	0.132248	2,24	2.20362
Fig. 1d Stressed OCR	0.811853	1,18	0.05835	0.20256	1,15	1.77577	0.281439	1,15	1.24838	0.422417	2,14	0.89346
Fig. 1d Baseline ECAR	0.578039	1,18	0.32094	0.969486	1,15	0.00151	0.590647	1,15	0.30212	0.80357	2,14	0.2207
Fig. 1d Stressed ECAR	0.609275	1,18	0.27059	0.113785	1,15	2.82024	0.22657	1,15	1.59012	0.238504	2,24	1.52249
Fig. 1d Met. Pot. OCR	0.139264	1,18	2.39322	0.139264	1,15	2.39322	0.564773	1,15	0.34666	0.18285	2,24	1.82526
Fig. 1d Met. Pot. ECAR	**0.061997**	1,18	3.96022	**0.00418**	1,15	11.38001	0.172928	1,15	2.04782	**0.009414**	2,24	5.7025

Figure 2

Values	all/old			all/AD			young/old			young/AD			old/AD			all groups		
	p-value	*F*		*p*-value	*F*		*p*-value	*F*		*p*-value	*F*		*p*-value	*F*		*p*-value	*F*	
Fig. 2a ATP	0.547382	1,25	0.37207	**0.006196**	1,28	8.76305	0.427554	1,18	0.6589	**0.037335**	1,21	4.9413	**0.013621**	1,15	7.80689	**0.013546**	3,46	3.96313
Fig. 2b Lactate	0.49256	1,25	0.48507	**0.00054**	1,28	15.26135	0.378525	1,18	0.81515	**0.007985**	1,21	8.59008	**0.000894**	1,15	17.04062	**0.000933**	3,46	6.4934
Fig. 2c glu	0.159688	1,16	2.17486	0.211074	1,25	1.64747	0.159688	1,16	2.17486	**0.078816**	1,20	3.43064	0.893954	1,12	0.01854	0.255139	3,41	1.40466
Fig. 2c IGF-1	**0.047218**	1,16	4.6213	**0.056848**	1,25	3.98708	**0.047218**	1,16	4.6213	**0.011787**	1,20	7.6787	0.775181	1,12	0.08534	**0.033693**	3,41	3.18506

Values	all/all			young/young			old/old			AD/AD		
	p-value	*F*		*p*-value	*F*		*p*-value	*F*		*p*-value	*F*	
Fig. 2c glu/IGF-1	**0.014082**	1,34	6.70032	**0.009156**	1,24	8.03632	0.599975	1,8	0.29809	0.203184	1,16	1.76054

Ratios	AD/old - AD/young			AD/old - old/young			AD/young - old/young			all groups		
	p-value	*F*		*p*-value	*F*		*p*-value	*F*		*p*-value	*F*	
Fig. 2a ATP	0.406568	1,18	0.72225	**0.005367**	1,15	10.57201	**0.022178**	1,15	6.50481	**0.014828**	2,24	5.04478
Fig. 2b Lactate	0.137797	1,18	2.4122	**0.000157**	1,15	25.03465	**0.000894**	1,15	17.04062	**0.000177**	2,24	12.65165
Fig. 2c glu	**0.013285**	1,16	7.7501	**0.054368**	1,12	4.54512	0.893954	1,15	0.01854	**0.02905**	2,20	4.24575
Fig. 2c IGF-1	**0.000312**	1,16	20.91642	**0.00465**	1,12	12.02547	0.775181	1,15	0.08534	**0.000243**	2,10	12.98618

Figure 3

Values	all/old			all/AD			young/old			young/AD			old/AD			all groups		
	p-value	*F*		*p*-value	*F*		*p*-value	*F*		*p*-value	*F*		*p*-value	*F*		*p*-value	*F*	
Fig. 3a NADt	0.658008	1,25	0.20071	**0.069448**	1,27	3.57456	0.524097	1,18	0.4221	0.123928	1,20	2.57953	**0.036667**	1,14	5.33489	0.223454	3,43	1.5179
Fig. 3b NADH	0.59242	1,25	0.29408	**0.05232**	1,27	4.15076	0.64844	1,18	0.431179	**0.092316**	1,20	3.12579	**0.028723**	1,14	5.9417	0.178992	3,43	1.71074
Fig. 3c NAD+	0.792168	1,25	0.07093	0.135894	1,27	2.36283	0.716928	1,18	0.13566	0.222359	1,20	1.58628	**0.07485**	1,14	3.70397	0.386044	3,43	1.03644
Fig. 3d ratios NAD+/NADH							0.923745	1,18	0.00942	0.328384	1,20	1.00131	0.412244	1,14	0.71135	0.429081	3,43	0.94127

Ratios	AD/old - AD/young			AD/old - old/young			AD/young - old/young			all groups		
	p-value	*F*		*p*-value	*F*		*p*-value	*F*		*p*-value	*F*	
Fig. 3a NADt	0.26741	1,18	1.30987	**0.021197**	1,15	6.62168	**0.040956**	1,15	5.00075	**0.009933**	2,24	5.62339
Fig. 3b NADH	0.165247	1,18	2.09215	**0.016968**	1,15	7.20818	**0.032312**	1,15	5.56456	**0.006346**	2,24	6.29383
Fig. 3c NAD+	0.62969	1,18	0.24061	**0.046076**	1,15	4.7287	**0.081443**	1,15	3.4889	**0.045177**	2,24	3.53359

Figure 4

Values	all/old			all/AD			young/old			young/AD			old/AD			all groups		
	p-value	*F*		*p*-value	*F*		*p*-value	*F*		*p*-value	*F*		*p*-value	*F*		*p*-value	*F*	
Fig. 4a mtMinArc	0.273096	1,22	1.26355	0.795938	1,25	0.06832	0.167003	1,16	2.09593	0.444566	1,19	0.60958	0.127131	1,13	2.65612	0.496459	3,41	0.80853
Fig. 4b citrate synthase	**0.080535**	1,24	3.32917	0.71023	1,26	0.14111	**0.009759**	1,17	8.4666	0.145599	1,19	2.30286	**0.078564**	1,14	3.60093	**0.063194**	3,42	2.61603
Fig. 4c MitoTracker	0.296862	1,20	1.4735	0.909028	1,22	0.01336	0.125382	1,14	2.65697	0.396134	1,16	0.76029	0.270792	1,12	1.33274	0.421599	3,36	0.96113

Ratios	AD/old - AD/young			AD/old - old/young			AD/young - old/young			all groups		
	p-value	*F*		*p*-value	*F*		*p*-value	*F*		*p*-value	*F*	
Fig. 4a mtMinArc	**0.022781**	1,16	6.34586	**0.021267**	1,13	6.85391	0.127131	1,13	2.65612	**0.006757**	2,21	6.39966
Fig. 4b citrate synthase	**0.001903**	1,16	13.76197	**0.078564**	1,14	3.60093	**0.000104**	1,14	28.51785	**0.000054**	2,22	15.85914
Fig. 4c MitoTracker	**0.016057**	1,14	7.48982	**0.008195**	1,12	9.99647	0.270792	1,12	1.33274	**0.007367**	2,19	6.43008

Figure 5

Values	all/old			all/AD			young/old			young/AD			old/AD			all groups		
	p-value	*F*		*p*-value	*F*		*p*-value	*F*		*p*-value	*F*		*p*-value	*F*		*p*-value	*F*	
Fig. 5a mtMajArc	0.261658	1,22	1.32726	0.605477	1,25	0.27369	0.152764	1,16	2.25378	0.32371	1,19	1.03674	0.982132	1,13	0.00052	0.439456	3,41	0.92049
Fig. 5c MTND4	0.97254	1,25	0.00121	**0.052942**	1,28	4.08416	0.959986	1,18	0.00259	**0.049733**	1,21	4.33587	0.12632	1,15	2.62051	0.249527	3,46	1.41828
Fig. 5d ROS	0.454524	1,25	0.57718	0.901284	1,28	0.01567	0.296998	1,18	1.15354	0.75182	1,21	0.10267	0.39652	1,15	0.76181	0.745131	3,46	0.41203
Fig. 5e MitoSox	0.193546	1,20	1.81016	0.701845	1,22	0.15044	**0.059758**	1,14	4.10581	0.331683	1,16	1.00219	0.496764	1,12	0.49119	0.343737	3,36	1.14595

Values	young			old			AD			all groups		
	p-value	*F*		*p*-value	*F*		*p*-value	*F*		*p*-value	*F*	
Fig. 5b ratios Min/MajArc	0.732633	1,16	0.12086	0.703651	1,19	0.14914	0.982132	1,13	0.00052	0.883234	2,24	0.12481

Ratios	AD/old - AD/young			AD/old - old/young			AD/young - old/young			all groups		
	p-value	*F*		*p*-value	*F*		*p*-value	*F*		*p*-value	*F*	
Fig. 5a mtMajArc	**0.056615**	1,16	4.22183	**0.044662**	1,13	4.93685	0.313243	1,13	1.10062	**0.027781**	2,21	4.27079
Fig. 5c MTND4	0.961343	1,18	0.00242	0.133851	1,15	2.51177	0.12632	1,15	2.62051	0.141882	2,24	2.12063
Fig. 5d ROS	0.206554	1,18	1.71701	**0.056325**	1,15	4.2772	0.39652	1,15	0.76181	0.133176	2,24	2.19534
Fig. 5e MitoSox	0.161721	1,14	2.18258	**0.02801**	1,12	6.2408	0.496764	1,12	0.49119	**0.095828**	1,19	2.66003

Figure 6																		
Values	all/old			all/AD			young/old			young/AD			old/AD			all groups		
	***p*-value**		***F***	***p*-value**		***F***	***p*-value**		***F***	***p*-value**		***F***	***p*-value**		***F***	***p*-value**		***F***
Fig. 6a PFKFB3	0.376775	1,23	0.81232	0.559347	1,26	0.34978	0.26475	1,17	1.33008	0.35001	1,20	0.91579	0.517872	1,13	0.4418	0.596567	3,43	0.63494
Fig. 6a LDH	0.301566	1,23	1.11688	0.676208	1,26	0.17842	0.19165	1,17	1.84911	0.37822	1,20	0.81141	0.162777	1,13	2.18949	0.511262	3,43	0.78066
Fig. 6b NMNAT2	0.480827	1,23	0.51354	0.632242	1,26	0.23453	0.350661	1,17	0.92101	0.958193	1,20	0.00282	0.157315	1,13	2.25222	0.722533	3,43	0.44434
Fig. 6b NAMPT	0.846593	1,23	0.03828	0.221133	1,26	1.56872	0.802091	1,17	0.06482	0.228427	1,20	1.53915	0.300545	1,13	1.15567	0.734866	3,43	0.4267
Fig. 6b PARP1	0.989364	1,22	0.00018	0.16267	1,25	2.06953	0.984552	1,16	0.00039	0.147933	1,19	2.2749	0.29834	1,13	1.17364	0.540827	3,41	0.72863
Fig. 6b SIRT1	0.471141	1,25	0.53542	0.386216	1,28	0.77484	0.348102	1,18	0.92818	0.259095	1,21	1.34544	0.99503	1,15	0.00004	0.593704	3,46	0.63913
Fig. 6b SIRT3	0.774264	1,25	0.08406	0.561469	1,27	0.34565	0.712509	1,18	0.14015	0.522698	1,20	0.4233	0.712245	1,14	0.1417	0.913815	3,45	0.17343
Fig. 6c IDH3A	0.163564	1,23	2.07131	0.5429	1,27	0.37969	**0.074048**	1,17	3.62327	0.949606	1,21	0.00409	**0.083126**	1,14	3.48207	0.309398	3,44	1.23214
Fig. 6c OGHD	0.112796	1,23	2.71832	0.643661	1,26	0.21905	**0.020241**	1,17	6.55939	0.553531	1,20	0.36316	**0.016819**	1,13	7.51396	**0.089023**	3,43	2.31692
Fig. 6c MDH1	0.672473	1,23	0.18337	0.615738	1,26	0.25806	0.569063	1,17	0.33721	0.802017	1,20	0.06456	0.442727	1,13	0.62683	0.946253	3,42	0.12262
Fig. 6c MDH2	0.670145	1,21	0.18662	0.663165	1,26	0.1941	0.618493	1,16	0.2579	0.57057	1,21	0.33207	0.648287	1,13	0.21801	0.898213	3,41	0.19645

Ratios	AD/old - AD/young			AD/old - old/young			AD/young - old/young			all groups		
	***p*-value**		***F***	***p*-value**		***F***	***p*-value**		***F***	***p*-value**		***F***
Fig. 6a PFKFB3	**0.051921**	1,16	4.41085	**0.041281**	1,13	5.12826	0.517872	1,13	0.4418	**0.035768**	2,21	3.91953
Fig. 6a LDH	**0.002451**	1,16	12.88749	**0.001832**	1,13	15.1959	0.162777	1,13	2.18949	**0.00034**	2,21	11.96584
Fig. 6b NMNAT2	0.119243	1,16	2.70962	**0.021282**	1,13	6.85193	0.157581	1,13	2.2491	**0.032876**	2,21	4.03582
Fig. 6b NAMPT	0.603268	1,18	0.27984	0.481704	1,14	52244	0.300545	1,14	1.15567	0.507036	2,23	0.69963
Fig. 6b PARP1	0.976	1,16	0.00093	0.306346	1,13	1.13381	0.29834	1,13	1.17364	0.410849	2,21	0.92829
Fig. 6b SIRT1	**0.092617**	1,18	3.15466	0.158986	1,15	2.19696	0.99503	1,15	0.00004	0.160051	2,24	1.97955
Fig. 6b SIRT3	0.313629	1,18	1.0824	0.732078	1,14	0.122	0.712245	1,14	0.1417	0.702811	2,22	0.35838
Fig. 6c IDH3A	**0.037418**	1,18	5.04914	**0.012477**	1,14	8.20792	**0.083126**	1,14	3.48208	**0.017106**	2,22	4.92265
Fig. 6c OGHD	**0.000505**	1,16	18.8484	**0.000214**	1,13	25.7212	**0.016819**	1,13	7.51396	**0.000055**	2,21	16.23784
Fig. 6c MDH1	0.452315	1,16	0.59345	0.199147	1,13	1.83036	0.442727	1,13	0.62683	0.373622	2,21	1.03214
Fig. 6c MDH2	**0.024422**	1,18	6.03357	**0.037884**	1,13	5.34026	0.648287	1,13	0.21801	**0.022292**	2,22	4.54392

Supplementary Table 2 | Statistical data using one-way analysis of variance (ANOVA) tests for independent measures.

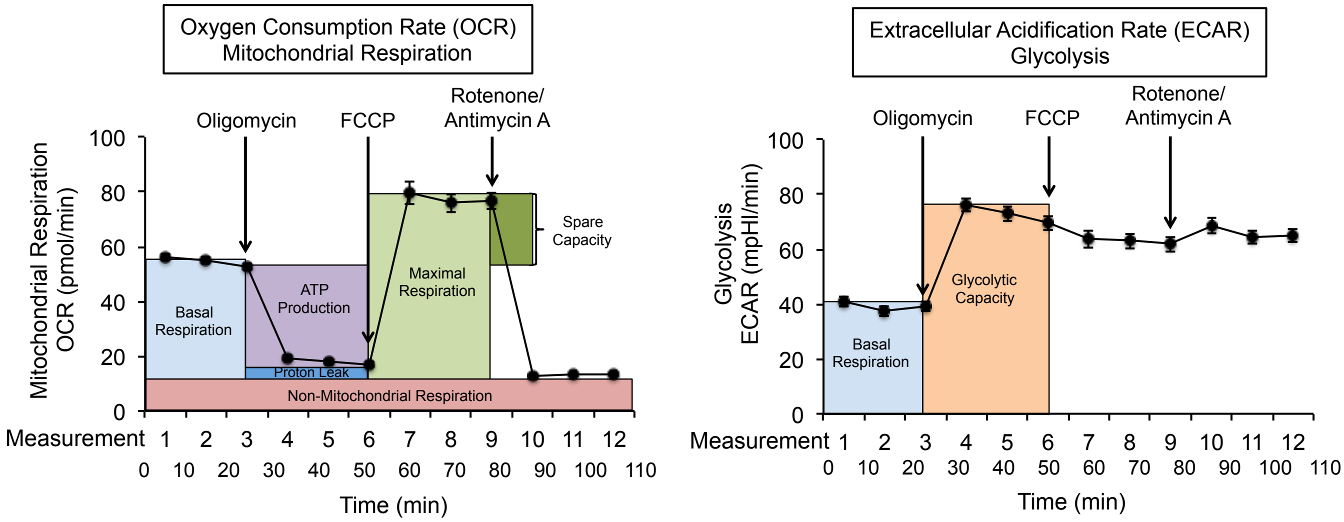

Supplementary Figure 1 | Schematic representation of Seahorse experiments-derived data. Profiles for oxygen consumption rate (OCR) and extracellular acidification rate (ECAR) with arrows indicating injections of specific pharmacologic stressors targeting the electron transport chain (ETC) and ATP production: Oligomycin, which inhibits complex IV (ATP synthase), decreasing OCR and ATP production; Carbonyl cyanite-4 (trifluoromethoxy) phenylhydrazone (FCCP), which disrupts the mitochondrial membrane potential and collapses the proton gradient at the ETC leading to maximal respiration (O_2 consumption by complex IV); and Rotenone/Antimycin A, which inhibit complex I and III resulting in the shut down of mitochondrial respiration. From the data generated, several additional measures can be calculated, including the spare respiratory capacity (maximal respiration minus basal respiration), the proton leak, non-mitochondrial respiration, and the coupling effect, which determines ATP production relative to basal respiration. The assay deducts the ECAR is an indicator of the cells' glycolytic capacity, and the proton production rate (PPR). Images are adapted from Seahorse Agilent Technologies.

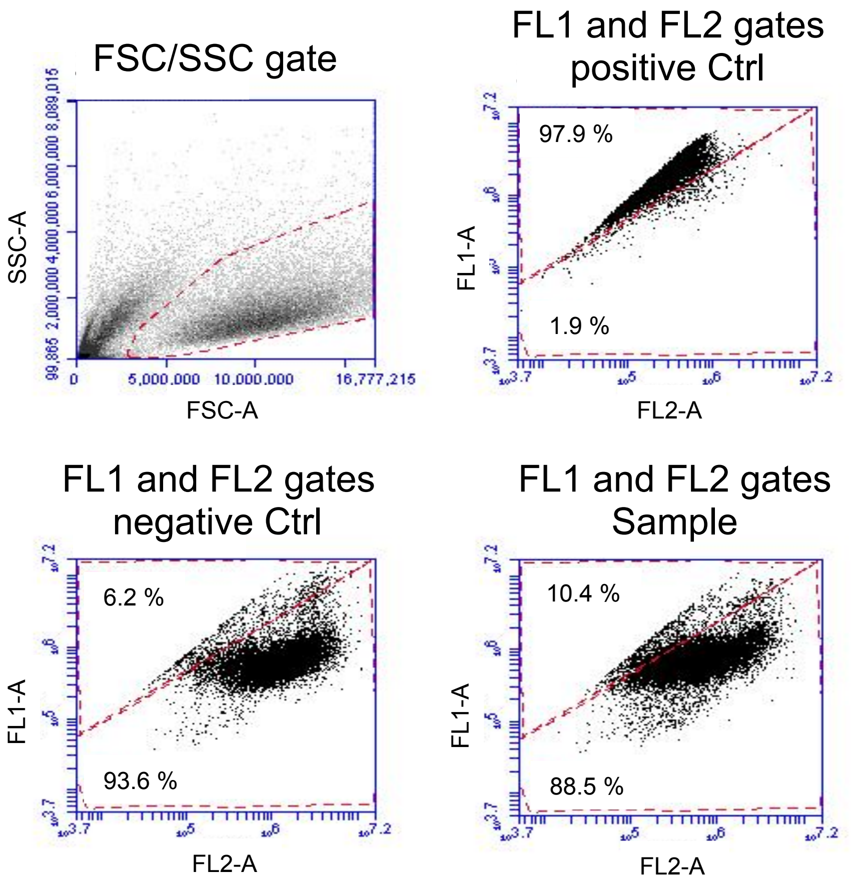

Supplementary Figure 2 | Gating strategy in the MitoPT JC-1 FCM assays. For positive and negative controls cells were treated with 50 µM of the depolarizing agent Carbonyl cyanide m-chlorophenyl hydrazone (CCCP) in DMSO or DMSO alone, respectively.

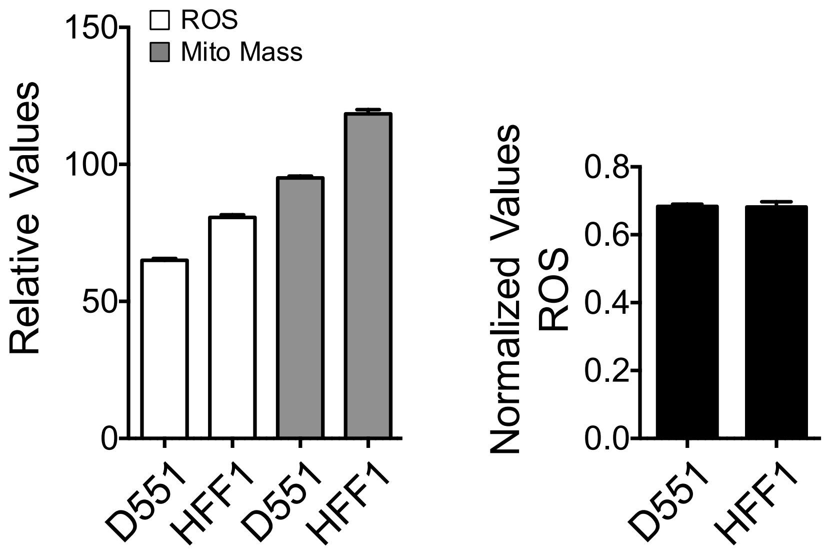

Supplementary Figure 3 | MitoSox and MitoTracker assay controls. Human foreskin cell line HFF1 and human fetal skin fibroblast cell line D551 were used as positive controls in combined MitoSox/MitoTracker assays measuring red (MitoSox) and green (MitoTracker) fluorescence in a BioTek plate reader. (**a**) Relative values of ROS (MitoSox) and mitochondrial mass (MitoTracker) after protein normalization with a BioRad Protein assay. (**b**) Both cell lines show similar levels of ROS after normalizing the MitoSox data with MitoTracker values.

Supplementary References

1 Salehi, M. H. *et al.* Gene expression profiling of mitochondrial oxidative phosphorylation (OXPHOS) complex I in Friedreich ataxia (FRDA) patients. *PLoS One* **9**, e94069, doi:10.1371/journal.pone.0094069 (2014).

2 Lupberger, J. *et al.* Quantitative analysis of beta-actin, beta-2-microglobulin and porphobilinogen deaminase mRNA and their comparison as control transcripts for RT-PCR. *Mol Cell Probes* **16**, 25-30, doi:10.1006/mcpr.2001.0392 (2002).

3 Li, H. *et al.* MiR-34b-3 and miR-449a inhibit malignant progression of nasopharyngeal carcinoma by targeting lactate dehydrogenase A. *Oncotarget* **7**, 54838-54851, doi:10.18632/oncotarget.10761 (2016).

4 Yalcin, A. *et al.* 6-Phosphofructo-2-kinase (PFKFB3) promotes cell cycle progression and suppresses apoptosis via Cdk1-mediated phosphorylation of p27. *Cell Death Dis* **5**, e1337, doi:10.1038/cddis.2014.292 (2014).

5 Di Stefano, M., Galassi, L. & Magni, G. Unique expression pattern of human nicotinamide mononucleotide adenylyltransferase isozymes in red blood cells. *Blood Cells Mol Dis* **45**, 33-39, doi:10.1016/j.bcmd.2010.04.003 (2010).

6 Soncini, D. *et al.* Nicotinamide phosphoribosyltransferase promotes epithelial-to-mesenchymal transition as a soluble factor independent of its enzymatic activity. *J Biol Chem* **289**, 34189-34204, doi:10.1074/jbc.M114.594721 (2014).

7 Dziaman, T. *et al.* PARP-1 expression is increased in colon adenoma and carcinoma and correlates with OGG1. *PLoS One* **9**, e115558, doi:10.1371/journal.pone.0115558 (2014).

8 Duncan, M. T. *et al.* SIRT1 is a critical regulator of K562 cell growth, survival, and differentiation. *Exp Cell Res* **344**, 40-52, doi:10.1016/j.yexcr.2016.04.010 (2016).

9 Lee, S. M. *et al.* Cytosolic malate dehydrogenase regulates senescence in human fibroblasts. *Biogerontology* **13**, 525-536, doi:10.1007/s10522-012-9397-0 (2012).

10 Snezhkina, A. V. *et al.* Differential expression of alternatively spliced transcripts related to energy metabolism in colorectal cancer. *BMC Genomics* **17**, 1011, doi:10.1186/s12864-016-3351-5 (2016).
